# Irinotecan-gut microbiota interactions and the capability of probiotics to mitigate Irinotecan-associated toxicity

**DOI:** 10.1186/s12866-023-02791-3

**Published:** 2023-03-02

**Authors:** Marwa S. Mahdy, Ahmed F. Azmy, Tarek Dishisha, Wafaa R. Mohamed, Kawkab A. Ahmed, Ahmed Hassan, Sahar El Aidy, Ahmed O. El-Gendy

**Affiliations:** 1 Microbiology and Immunology Department, Faculty of Pharmacy, Beni-Suef University, Salah Salem Street, Beni-Suef, 62511 Egypt; 2 Department of Pharmacology and Toxicology, Faculty of Pharmacy, Beni-Suef University, Beni-Suef, Egypt; 3 Pathology Department, Faculty of Veterinary Medicine, Cairo University, Giza, 12211 Egypt; 4 Department of Clinical Oncology, Faculty of Medicine, Beni-Suef University, Beni-Suef, Egypt; 5 Host-Microbe Interactions, Groningen Biomolecular Sciences and Biotechnology Institute (GBB), University of Groningen, Groningen, The Netherlands

**Keywords:** Irinotecan, Anticancer, Beta-glucuronidase, Probiotics, Colon, Inflammation

## Abstract

**Background:**

Irinotecan is a chemotherapeutic agent used to treat a variety of tumors, including colorectal cancer (CRC). In the intestine, it is transformed into SN-38 by gut microbial enzymes, which is responsible for its toxicity during excretion.

**Objective:**

Our study highlights the impact of Irinotecan on gut microbiota composition and the role of probiotics in limiting Irinotecan-associated diarrhea and suppressing gut bacterial β-glucuronidase enzymes.

**Material and methods:**

To investigate the effect of Irinotecan on the gut microbiota composition, we applied *16S rRNA* gene sequencing in three groups of stool samples from healthy individuals, colon cancer, and Irinotecan treated patients (*n* = 5/group). Furthermore, three *Lactobacillus* spp.; *Lactiplantibacillus plantarum (L. plantarum)*, *Lactobacillus acidophilus (L. acidophilus)*, *Lacticaseibacillus rhamnosus* (*L. rhamnosus)* were used in a single and mixed form to *in-vitro* explore the effect of probiotics on the expression of β-glucuronidase gene from *E. coli*. Also, probiotics were introduced in single and mixed forms in groups of mice before the administration of Irinotecan, and their protective effects were explored by assessing the level of reactive oxidative species (ROS) as well as studying the concomitant intestinal inflammation and apoptosis.

**Results:**

The gut microbiota was disturbed in individuals with colon cancer and after Irinotecan treatment. In the healthy group, *Firmicutes* were more abundant than *Bacteriodetes,* which was the opposite in the case of colon-cancer or Irinotecan treated groups. *Actinobacteria* and *Verrucomicrobia* were markedly present within the healthy group, while *Cyanobacteria* were noted in colon-cancer and the Irinotecan-treated groups. *Enterobacteriaceae* and genus *Dialister* were more abundant in the colon-cancer group than in other groups. The abundance of *Veillonella*, *Clostridium*, *Butryicicoccus,* and *Prevotella* were increased in Irinotecan-treated groups compared to other groups. Using *Lactobacillus* spp. mixture in mice models significantly relieved Irinotecan-induced diarrhea through the reduction of both β-glucuronidase expression and ROS, in addition to guarding gut epithelium against microbial dysbiosis and proliferative crypt injury.

**Conclusions:**

Irinotecan-based chemotherapy altered intestinal microbiota. The gut microbiota participates greatly in determining both the efficacy and toxicity of chemotherapies, of which the toxicity of Irinotecan is caused by the bacterial ß-glucuronidase enzymes. The gut microbiota can now be aimed and modulated to promote efficacy and decrease the toxicity of chemotherapeutics. The used probiotic regimen in this study lowered mucositis, oxidative stress, cellular inflammation, and apoptotic cascade induction of Irinotecan.

**Supplementary Information:**

The online version contains supplementary material available at 10.1186/s12866-023-02791-3.

## Introduction

Pharmacomicrobiomics studies the interactions between pharmaceuticals and the human gut microbiota. Drug bioavailability, clinical effectiveness, and toxicity can all be affected by the gut microbes and their enzymatic products through both direct and indirect mechanisms [1].

Irinotecan (CPT-11) is derived from a camptothecin alkaloid, which has cytotoxicity in various tumors such as colorectal, pancreatic, and lung cancer. Irinotecan is a prodrug of the active form SN-38 that hinders the enzyme topoisomerase-I that is included in the replication of DNA. SN-38, a more potent metabolite of Irinotecan, is released from hepatocytes' CES1 and CES2 carboxylesterases after hydrolysis. SN-38 has hundreds to thousands of times the cytotoxicity of Irinotecan until it is enzymatically converted in the hepatic cells into inactive SN-38 glucuronide (SN-38G) through the action of uridine diphosphate glucuronosyltransferases (UGT), which conjugates SN-38 with glutaric acid, allowing it to be excreted in the intestines with the bile [2].

Morbidity and mortality during Irinotecan-based chemotherapy could result from severe diarrhea due to the effect of gut bacterial enzymes. β-D-glucuronidase (GUSs) is a gut-bacterial enzyme that deconjugates SN-38G in the intestinal lumen releasing the toxic form SN-38, the primary cause of diarrhea. These toxic effects have been demonstrated to be reduced by selective inhibitors of gut bacterial GUS [3, 4]. Other adverse effects of Irinotecan include leukopenia and mucositis. Chemotherapy-induced mucositis (damage to the mucosal barrier) is a serious oncological issue that manifests in many clinical symptoms such as nausea, vomiting, diarrhea, and weight loss. Moreover, facultative anaerobes and opportunistic microbes such as *Enterococcus* spp., *Streptococcus* spp., *Staphylococcus* spp., and *Enterobacteriaceae* from the intestines of CPT-11-treated patients could translocate and cause sepsis [5, 6]. Irinotecan-induced mucositis is caused by activating inflammatory pathways that release reactive oxygen species (ROS), leading to protein damage, DNA mutations, oxidation of membrane phospholipids, and alteration of low-density lipoproteins [7].

Evidences propose that alteration of the intestinal bacteria could participate significantly in the cancer evolution [8–12]. Bacteria can aid in the cancer beginning and progress through various mechanisms. The gut microbial changes can enhance opportunistic pathogenic microorganisms and lead to a chronic inflammation that is a result of increased mucosal permeability, which allows bacteria and their products to enter the body and activate both the natural and acquired immunological responses [13–15]. Toxins and metabolites from translocated bacteria can influence the DNA stability, cell cycle, cell proliferation, as well as tumor initiation and development [3, 16].

Probiotics can mitigate the effects of gut microbiome modification. Probiotics are bacteria that are used as medicines or food supplements; they serve to sustain a healthy microbiological equilibrium in the GIT of humans and other animals. In addition, probiotic bacteria could suppress intestinal β-D-glucuronidase function [17], indicating that they may be used to avoid anticancer-induced diarrhea in cancer patients receiving Irinotecan.

The hypothesis that probiotics might serve as potential inhibitors of β-D-glucuronidase was the focus of the current study to propose a supportive probiotic regimen to counteract Irinotecan toxicity. Also, the purpose of this study was to shed light on the influence of Irinotecan on the gut microbial community to identify potential dysbiosis and assess the impacts on colon epithelial barrier integrity and inflammation. In addition, we investigated the impact of probiotics in suppressing oxidative stress or boosting natural antioxidant defenses *in-vivo* for prospective use to overcome Irinotecan-induced diarrhea.

## Materials and method

### Bacterial strains and growth conditions

Three clinical *E.coli* isolates, recovered from stool samples of healthy volunteers, were kindly provided from the Microbiology and Immunology Department, Faculty of Pharmacy, Beni-Suef University. The probiotic *Lactobacillus* spp. used in our study and bacterial culture conditions are shown in (Table 1). Each bacteria was maintained in 25% glycerol and stored at -20 °C.

**Table 1:** The bacterial strains used in this study, their growth media and growth conditions

Bacterial strains	Culture Media	Growth Temperature
*Lactiplantibacillus plantarum* W21	MRS^a^	30 °C
*Lactobacillus acidophilus* W22	MRS	30 °C
*Lacticaseibacillus rhamnosus* W184	MRS	30 °C
*E. coli* AF01	MacConkey/BHI^b^	37 °C
*E. coli* AF02	MacConkey/BHI	37 °C
*E. coli* AF03	MacConkey/BHI	37 °C

^a^MRS: De Man, Rogosa and Sharpe agar

^b^BHI: Brain heart infusion

### Phenotypic screening of *E. coli* for β-glucuronidase

Detection of β-glucuronidase was performed in presence of p-nitrophenyl-p-D-glucopyranosiduronic acid (PNPG) as a chromogenic enzyme substrate. The PNPG discs were prepared by impregnating discs with 50 µl of a 1% PNPG solution in 100 mM phosphate buffer, pH 8 [18]. The prepared discs were allowed to dry overnight at 36 °C and then maintained cool in vials at 4 °C in the existence of a desiccator.

*E. coli* suspension (4 McFarland standard) was prepared in sterile distilled water from an overnight BHI agar plate and then cultured for 24 h at 37 °C in the presence of a disc saturated with the colorless substrate (PNPG). The test was monitored for 2, 6, and 24 h, and upon hydrolysis of the PNPG substrate, the yellow color of p-nitrophenol indicated a positive reaction. Also, negative controls were recruited at which the supernatant remained colorless.

### The growth kinetics of *E. coli* in the presence of Irinotecan at different concentrations

Irinotecan (Sigma Aldrich chemical company, St. Louis, Mo, USA) was prepared in a double-strength BHI broth to get final concentrations of 10, 5, 2.5, 1.25, 0.625, 0.312 mg/ml and spread in 96 wells microtiter plate as 100 μL per each well. The standardized inoculums of three *E. coli* isolates were prepared by diluting overnight growth of each isolate in 0.9% NaCl solution to obtain the turbid suspension of 0.5 McFarland standard used to inoculate wells of microtiter plate [19]. Positive growth control wells having bacteria without Irinotecan and negative control wells with BHI broth without bacteria were included. Before data processing, the plates were placed at 37 °C for 18 h with continuous shaking at 120 rpm. A microtiter plate reader (Tecan Sunrise, Austria) was used to measure the optical density (OD) of each well at 620 nm, and measurements were taken every 30 min interval [20]. Each treatment was performed in triplicates.

Based on the resulting data, growth curves and maximum growth kinetics at specific time points (µ_max_) were evaluated compared to positive growth controls. Normalization was done by subtracting all of the experiment outcomes from the background negative controls. The following formula was used to find µ_max,_ where X_t_; is the growth absorbance at a particular time point, X_0_; is the initial growth absorbance, and t; is the time at which µ_max_ is obtained.

$${\mathrm{X}}_{t} = {\mathrm{X}}_{0}\mathrm{ exp }\left({\upmu }_{\mathrm{max}} .\mathrm{ t}\right)$$

### Metagenomics analysis of whole gut microbiota in response to Irinotecan treatment

#### Ethical considerations

Ethical approval was obtained from Research Ethical Committee at the Faculty of Medicine, Beni-Suef University (FM-BSU REC), with approval number; FMBSUREC/05072020/Mahdy.

Guidelines of the Declaration of Helsinki, International Conference of Harmonization ICH, and United States Codes of Federal Regulations and registered under the Federal Wide Assurance (FWA) for the protection of Human Subjects were followed. Informed consent was obtained from all patients included in the study. Data would be confidential and anonymous. Sociodemographic questions were for identifying the characteristics not identity. The participants themselves would not be obliged to participate.

#### Design, samples collection, and DNA extraction

Fifteen volunteers were the source of stool samples analyzed in this study, and their metadata were reported (Table 2). Samples were taken from five healthy people, five colon cancer patients, and five colon cancer patients taking Irinotecan within one week of their last dose of Irinotecan. All patients and healthy individuals were adult males and females, aging between 19 and 41, and had not been administered antibiotics for at least 3 months before samples collection. Healthy subjects had no chronic or infectious diseases and no previous history of gastrointestinal disease. Stool samples were collected in sterilized containers and immediately stored at − 20 ℃ for further DNA extraction.

**Table 2:** Different groups at which stool samples were collected

Samples	Gender	Age	Subject	Job
Sample1	Female	27	Healthy	Teacher
Sample2	Male	30	Healthy	Accountant
Sample3	Male	23	Healthy	No
Sample4	Male	21	Healthy	Student
Sample5	Male	22	Healthy	Student
Sample6	Female	40	Colon_Cancer	No
Sample7	Female	41	Colon_Cancer	No
Sample8	Female	28	Colon_Cancer	No
Sample9	Male	27	Colon_Cancer	Driver
Sample10	Male	41	Colon_Cancer	Driver
Sample11	Male	19	Irinotecan	No
Sample12	Female	42	Irinotecan	No
Sample13	Male	40	Irinotecan	Farmer
Sample14	Male	31	Irinotecan	No
Sample15	Female	36	Irinotecan	No

DNA was extracted from stool samples using an Igenomic stool DNA Extraction mini kit **(**iNtRON Biotechnology, Korea). The manufacturer's instructions were followed precisely. DNA was initially tested for quality and quantity in a NanoDrop 2000 UV–Vis spectrophotometer (Thermo Fisher Scientific, Waltham, MA, USA).

#### 16S rRNA amplification of V3-V4 region and Illumina sequencing

DNA samples were submitted for *16S rRNA* gene sequencing at the Integrated Microbiome Resource (IMR) at Dalhousie University (Halifax, Canada). Variable regions V3-V4 of the bacterial *16S rRNA* gene were amplified from all purified DNA samples using a set of primers 341F: 5'- CCTACGGGNGGCWGCAG -3' and 805R: 5'- GACTACHVGGGTATCTAATCC -3' [21] and sequenced on an Illumina MiSeq using paired-end 300 bp sequencing [22, 23].

The 16S fusion primers were added to the multiplexed samples in equal amounts. Illumina Nextera adapters and barcodes were included in the fusion primers for dual-labeling at both ends of the amplicons. The reaction mixture of 25 µL contained 5 µL of 5 × HF buffer, 0.5 µL dNTPs (40 mM), 5 µL forward and 5 µL reverse primer (1 µM), 0.25 µL Phusion polymerase (2 U/µL; Thermo Scientific), 2 µL template and 7.25 µL water. The reaction conditions started with denaturation at 98 °C (30 s), followed by 30 cycles of 98 °C (10 s), 55 °C (30 s), and 72 °C (30 s). The final extension was performed for 4.5 min at 72 °C. The samples and negative controls were combined to form a single library and then applied to the Illumina MiSeq platform using 2 × 300 bp Pair-End v3 chemistry according to the manufacturer's protocol.

#### Bioinformatic analysis

The QIIME2 Core (2019.10) was implemented for sequence analysis and primary statistics (Bolyen et al., 2019). The QIIME 2 demux plugin was used to demultiplex raw FASTQ files based on their unique barcodes [24]. Chimeric sequences were identified and deleted from each demultiplexed sequences after quality filtering, trimming, and de-noising using the QIIME 2 deblur plugin to attain the feature table [25]. The feature sequences had been aligned to the GREENGENES 13_8 99% database via the QIIME 2 feature-classifier plugin, and the taxonomy table was generated for taxonomic assignment and analysis [26]. The data was rarefied before alpha and beta diversity analysis using a depth of 4400 reads. Diversity metrics were calculated and plotted using the alpha group significance core metrics plugin (Chao1 and Shannon) and the beta diversity ordination emperor plugin (Bray Curtis Index) within QIIME2 [27]. The differences in the relative abundance of taxa between the patients, treated, and healthy individuals were detected by the linear discriminant analysis effect size (LEfSe) [28], DESeq2 for differential analysis of count data [29] and the differential abundance analysis with ANCOM [30]. PICRUSt2 was applied to predict microbial metabolic pathways and assess potential functional implications [31]. MicrobiomeAnalyst web-based tool was used for comprehensive statistical, visual, and meta-analysis of microbiome data [32].

### Expression of β-glucuronidase in response to different probiotic treatments

The in vitro capability of *L. plantarum*, *L. acidophilus*, *L. rhamnosus,* or their mixture to alter the expression of β-glucuronidase was studied. Each *Lactobacillus* spp. was subcultured in 30 mL MRS broth (Himedia) and incubated overnight at 30 °C. The freshly grown culture was divided into 3 portions of 10 mL. Each part was centrifuged at 10,000 g for 5 min, and the bacterial pellets of one part were washed twice in 10 mL saline and re-centrifuged to collect pellets. The cell-free supernatant (CFS) of the second part was filter-sterilized using a 0.22 um filter, and its pH was recorded. The CFS of the third part was filter sterilized and adjusted to pH 7 using 0.1 M NaOH.

*E. coli* AF02 was allowed to grow at 30 °C for 18 h, whether separately or in a co-culture habitat with 1% *Lactobacillus* spp. pellets, crude CFS, or CFS adjusted to pH 7 of *L. acidophilus*, *L. plantarum*, *L. rhamnosus* or their mixture [33]. Generally, the CFS was added as 50% proportions (v/v) in 20 mL final volume of double-strength BHI (co-culture media). To prepare a mixture of metabolites from the three tested *Lactobacillus* spp., the CFS of each bacteria was mixed equally with an equal volume of double strength BHI to form 20 ml final volume.

### RNA extraction and β-glucuronidase expression

Genomic RNA was extracted from *E.coli* either untreated or treated with *Lactobacillus* spp*.* and their metabolites using the Fast Q RNA extraction kits (iNtRoN, Korea) according to the manufacturer's instructions. Real-time RT-PCR analysis was carried out using the DTlite real-time PCR instrument (DNA-Technology, Russia). The gene encoding β-glucuronidase was amplified using primers uidAF (5′-CAACGAACTGAACTGGCAGA-3′) and uidAR (5′-CATTACGCTGCGATGGAT3′) (Macrogen, Southern Korea) and uidAP-FAM (5′-CCCGCCGGGAATGGTGATTAC3′). The final volume of each reaction mixture was 10 μL and included 5 μL TOPrea ${\mathrm{l}}^{\mathrm{TM}}$ One-step RT qPCR kit (TaqMan Probe) (Enzynomics, Korea), 1 μL of each primer, 2.5 μL of genomic RNA (100 ng/μL), and 0.25 μL of probe, and 0.25 μL of nuclease-free water. Amplification included a reverse transcription step at 50 °C for 30 min, followed by initial denaturation at 95 °C for 10 min, and 30 repeated cycles of denaturation at 95 °C for 5 s, and annealing/elongation at 60 °C for 30 s. In addition, the *E.coli* reference gene was amplified using primer cysGF (5′-TTGTCGGCGGTGGTGATGT-3′) and cysGR (5′ATGCGGTGAACTGTGGAATAAA-3′) (Macrogen, Southern Korea).

The expression level of each gene was analyzed as suggested [34]. The change in expression of each gene was recorded as the fold change in expression (Fc). The results reflect a logarithmic fold increase relative to the control samples. The following formula was used to obtain fold change:

$${\mathrm{Fold\ change }= 2}^{-\Delta \Delta \mathrm{CT}}$$

Ct; the cycle threshold (Ct) of the sample, the symbol **∆** refers to delta.

$$\Delta \Delta \mathrm{Ct }= \Delta \mathrm{Ct }\left(\mathrm{treated\ sample}\right)- \Delta \mathrm{Ct }(\mathrm{untreated\ sample})$$

Essentially, ∆∆Ct is the difference between the ∆Ct values of the treated/experimental sample and the untreated/control sample:

$$\Delta \mathrm{Ct }=\mathrm{ Ct }\left(\mathrm{gene\ of\ interest}\right)-\mathrm{ Ct }\left(\mathrm{housekeeping\ gene}\right)$$

### Counteracting Irinotecan induced toxicity using probiotics in mice model

#### Animals

Adult male Swiss mice weighing (30 ± 2 g) were obtained from the animal house of NAHDA University, Beni-Suef, Egypt. Mice were acclimatized for seven days in the animal house prior to the experiment. They were maintained under standard housing conditions; room temperature 26 ± 2 °C, with 12/12 h light and dark cycles, with free access to food and water. Handling of animals and all experimental procedures were approved by the Ethics Committee for Animal Experimentation (Institutional Animal Care and Use Committee, Beni-Suef University) with approval number: 021–200, and the guidelines of the Guide for the Care and Use of Laboratory Animals (NIH publication No. 85–23).

#### Experimental design

A total of 60 Swiss mice were divided into 10 groups (*n* = 6) as follows:

- **Group I**: Normal control group received a vehicle once daily for 22 days.
- **Group II:** *L. acidophilus* control group received *L. acidophilus* (200 µl, p.o) once daily for 22 days.
- **Group III**: *L. plantarum* control group received *L. plantarum* (200 µl, p.o) once daily for 22 days.
- **Group IV**: *L. rhamnosus* control group received *L. rhamnosus* (200 µl, p.o) once daily for 22 days.
- **Group V:** Mixture control group received a mixture of the three *Lactobacillus* spp. (200 µl, p.o) once daily for 22 days.
- **Group VI**: Irinotecan control received vehicle once daily for 22 days + Irinotecan (270 mg/kg, i.p) once on the 21^st^ day.
- **Group VII**: Irinotecan + *L. acidophillus* group received *L. acidophillus* (200 µl, p.o) once daily for 22 days + Irinotecan once on the 21^st^ day.
- **Group VIII**: Irinotecan + *L. plantarum* group received (200 µl p.o) once daily for 22 days + Irinotecan once on the 21^st^ day.
- **Group IX**: Irinotecan + *L. rhamnosus* group received *L. rhamnosus* (200 µl p.o) once daily for 22 days + Irinotecan once on the 21^st^ day.
- **Group X:** Irinotecan + Mixture group received a mixture of the three *Lactobacillus* spp. (200 µl p.o) once daily for 22 days + Irinotecan once on the 21^st^ day.

After 48 h of Irinotecan injection, mice were sacrificed then colon was dissected out and gently washed using normal saline to get rid of any fecal residues. One part of the colon was immersed in 10% phosphate-buffered formalin solution for histopathological examination and immune-histochemical analysis of caspase-3. The other colon part was homogenized in 0.1 M phosphate buffer saline and then centrifuged (at 1000 × g) for 10 min at 4 ˚C. The resultant supernatant was then discarded and used for estimation of oxidative stress biomarkers, including colon contents of malondialdehyde (MDA), reduced glutathione (GSH), and superoxide dismutase (SOD) activity, and inflammatory biomarkers, including tumor necrosis factor- α (TNF-α) and interleukin-6 (IL-6) expression.

### Biochemical analysis

#### Assessment of oxidative stress biomarkers

Colon content of MDA, reduced GSH, and activity of SOD were measured calorimetrically using kits purchased from Biodiagnostic (Cairo, Egypt). MDA was expressed as nmol/g tissue, reduced GSH was expressed as mmol/g tissue, and SOD activity was expressed as U/g tissue.

#### Assessment of inflammatory biomarkers

The tissue expression level of IL-6 and TNF-α were measured using ELISA kits purchased from R and D systems (USA) and CUSABIO Biotech Co, Wuhan (China), respectively, according to the instructions of the manufacturer.

#### Histopathological analysis

Specimens from the colon of mice were collected, preserved in neutral buffered formalin 10%, and processed by paraffin embedding technique. Transverse sections of 4–5 μm thickness were prepared and stained with Haematoxylin and Eosin (H & E) [35] and inspected blindly by a pathologist under a light microscope (BX43, Olympus). Quantitative histopathological assessment of colon lesions was carried out and scored from (0–3) in five randomly checked microscopic fields per animal (*n* = 6) as follows: (0) showed no changes, (1), (2), and (3) showed mild, moderate and severe changes, respectively. Briefly, the assigned lesions were mucosal necrosis, mucosal inflammatory cells infiltration, submucosal edema, submucosal inflammatory cells infiltration, and apoptosis of mucosal and glandular epithelium [36].

#### Immunohistochemical analysis

##### Cleaved Caspase-3 expression

Cleaved Caspase-3 expression level in the colon tissue was examined according to [37]. Sections were placed with primary antibodies against caspase‑3 (cat. no. sc-7148; polyclonal rabbit cleaved caspase 3 antibodies; 1:100; Santa Cruz Biotechnology, Inc.) overnight at 4˚C. After incubation with the corresponding secondary antibody, goat anti-rabbit IgG-FITC (cat. no. sc-2012; 1:100; Santa Cruz Biotechnology, Inc.) was used. Diaminobenzidine tetrachloride (DAB, Sigma Chemical Co., St. Louis, MO, USA) was used to visualize the immune reaction. The positive immune reactive cells showed brown-stained cytoplasm. Staining intensity and its distribution were classified as negative (no staining), weak, moderate, or strong intensity. The amount of cleaved caspase-3 was determined by averaging the percent area expression of five randomly selected areas in each segment using image analysis software (Image J, version 1.46a, NIH, Bethesda, MD, USA).

#### Statistical analysis

The data in this study are recorded as mean ± SEM. Comparisons were made using one-way ANOVA followed by the Tukey–Kramer post hoc multiple comparisons test. Nonparametric data were analyzed using the Kruskal Wallis test followed by the Dunn test and was expressed as median and interquartile range. Graph Pad Prism Program, v.5. was implemented for analyzing data. *P* < 0.05 are considered statistically significant.

## Results

### Screening of *E. coli* for β-glucuronidase activity and their growth kinetics in response to different concentrations of Irinotecan

In this study, the three clinical *E.coli* isolates, recovered from stool samples of healthy volunteers, were phenotypically screened for their capability to produce β-glucuronidase enzyme, which is responsible for the metabolic transformation and toxicity of Irinotecan. The three tested *E. coli* were able to hydrolyze the p-nitrophenyl-p-D-glucopyranosiduronic acid (PNPG), a chromogenic enzyme substrate, and produced canary yellow color after 6 h, indicating the *β-glucuronidase* enzymatic activity.

**Fig. 1:**
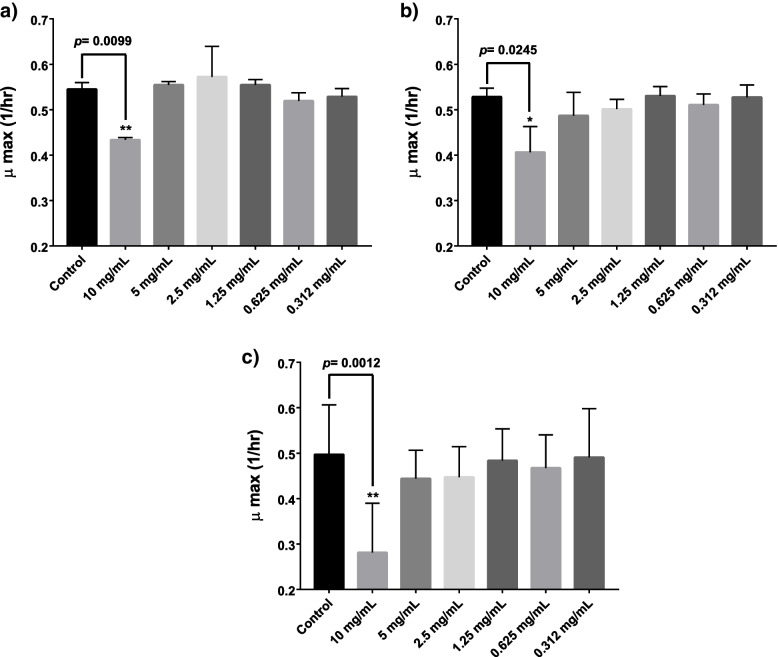
Effect of different Irinotecan concentrations on the growth kinetics of three selected *E.coli* isolates; (**A**) *E. coli* AF01, (**B**) *E. coli* AF02, and (**C**) *E. coli* AF03**.** * *p* < 0.05 ** *p* < 0.01

To estimate the possible inhibitory impact of Irinotecan on the growth kinetics of *E. coli*, Irinotecan was administered at different concentrations, and it showed no effects on the growth kinetics of *E. coli* except at its highest tested concentration of 10 mg/mL when compared to positive growth control groups. Irinotecan (10 mg/mL) significantly delayed the growth kinetics of *E. coli* without causing complete growth inhibition, as seen in (Fig. 1).

### Alteration of gut microbiota in colon cancer and Irinotecan treated patients when compared to healthy group

**Fig. 2:**
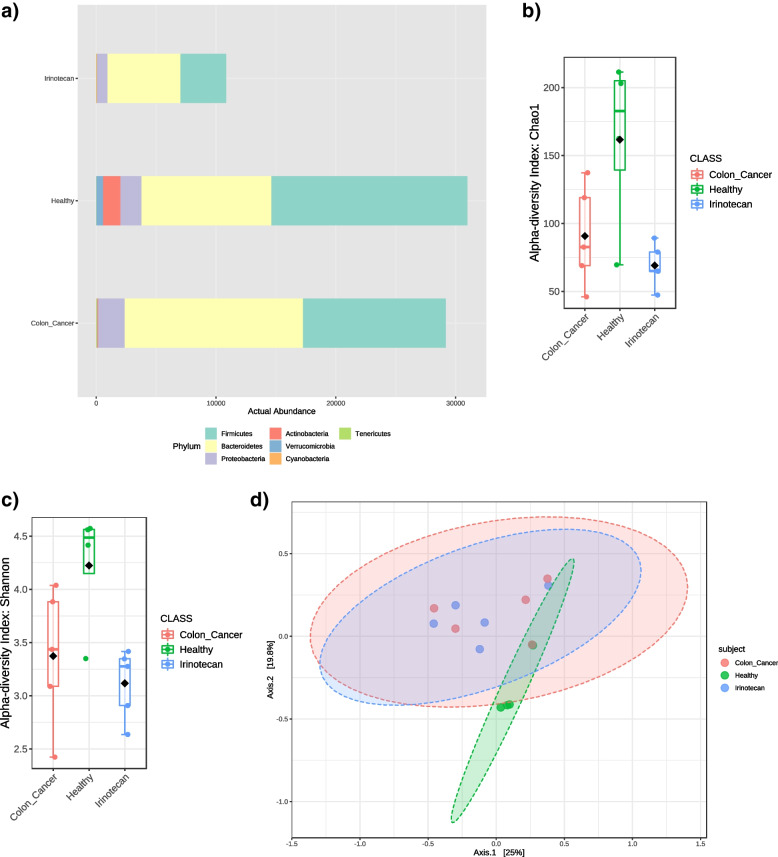
The gut microbiota actual phylum abundance in healthy, colon-cancer, and Irinotecan groups, as assessed by *16S rRNA* metagenomics sequencing. Irinotecan lowered the microbiota actual abundance (**a**). Alpha diversity estimation in healthy, colon cancer, and Irinotecan treated groups using (**b**) Shao index showing a significant diversity with *p*-value 0.019025 [ANOVA] F-value 5.8035, (**c**) Shannon index showing a significant diversity with *p*-value 0.027945 [ANOVA] F-value 5.0403. Beta diversity among three groups based on Bray Curtis Index, [PERMANOVA] F-value 1.6378; R-squared 0.22945; *p*-value 0.032 (**d**)

The gut microbiota composition in the three groups of healthy individuals, colon cancer and Irinotecan treated patients, were analyzed by a *16S rRNA* metagenomics sequencing approach. Colon-cancer and Irinotecan treatment caused an obvious microbiota perturbation compared to the healthy group (Fig. 2a) and (Fig. S1). In the healthy group, *Firmicutes* were more abundant than *Bacteriodetes*, which was the opposite in colon-cancer or Irinotecan treated groups. *Actinobacteria* and *Verrucomicrobia* were markedly present within the healthy group, while *Cyanobacteria* were noted in colon-cancer or Irinotecan treated groups.

Several alpha diversity indices (e.g., Chao or Shannon) were used to show the richness and diversity of gut microbiota in the Irinotecan treated group were decreased compared to the healthy and colon-cancer groups (Fig. 2b, c). Core bacterial taxa at each group were identified at different taxonomic levels (Fig. S2).

Beta diversity and principal coordinate analysis (PCoA) based on Bray Curtis Index was performed to uncover differences in the structure of gut microbiota across all groups based on the relative abundance of OTUs. The data showed significant dissimilarity between the healthy group and other groups' communities (*p* < 0.05). However, both colon-cancer and Irinotecan groups were more or less similar (Fig. 2d).

**Fig. 3:**
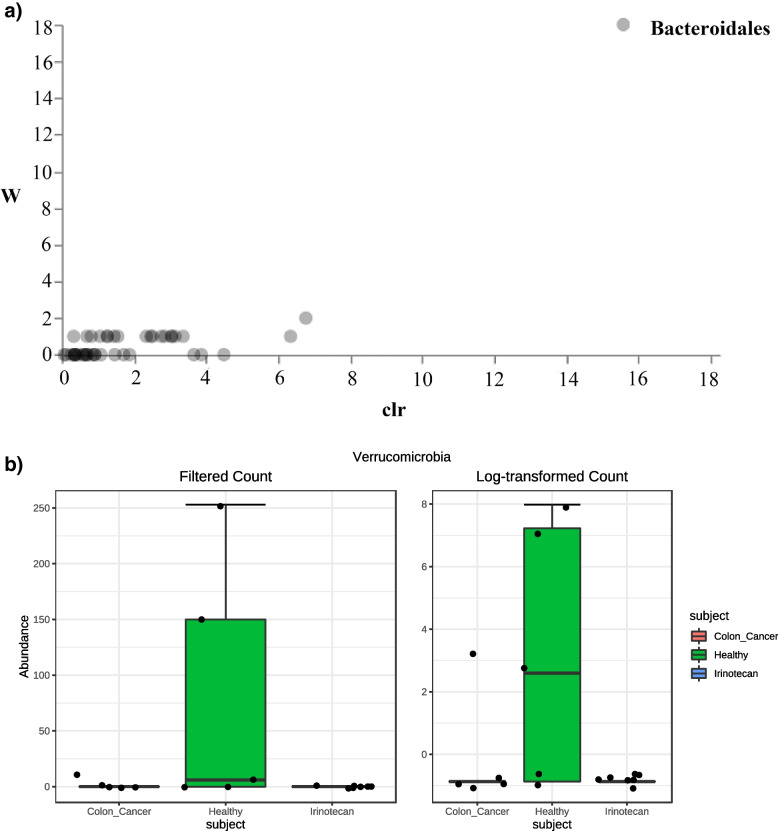
Differential abundance using (**a**) ANCOM analysis plot, showing the abundance of *Bacteroidales* in the healthy group, and using (**b**) DESeq2 analysis, showing the phylum *Verrucomicrobia* as the most significant marker in the healthy group

Differential abundance testing involves the use of statistical testing to determine if the relative abundances of certain microorganisms are significantly different between the study groups. ANCOM could identify taxa that are present in different abundances across sample groups by comparing the log ratio of each taxon's abundance to that of all remaining taxa one at a time. ANCOM analysis showed that *Bacteroidales* could represent a biomarker for healthy groups than other groups (Fig. 3a). Moreover, differential abundance analysis using DESeq2 was conducted, and it showed that the phylum *Verrucomicrobia* was more significant in the healthy group than in other groups (Fig. 3b). Correlation Network and the pattern search plot of top features of microbiota in healthy, colon-cancer, and Irinotecan groups are represented in (Fig. S3 and S4), respectively. Moreover, phylogeny and abundance based dendrogram of the microbiota in healthy, colon-cancer, and Irinotecan groups at different taxonomic levels are represented in (Fig. S5).

**Fig. 4:**
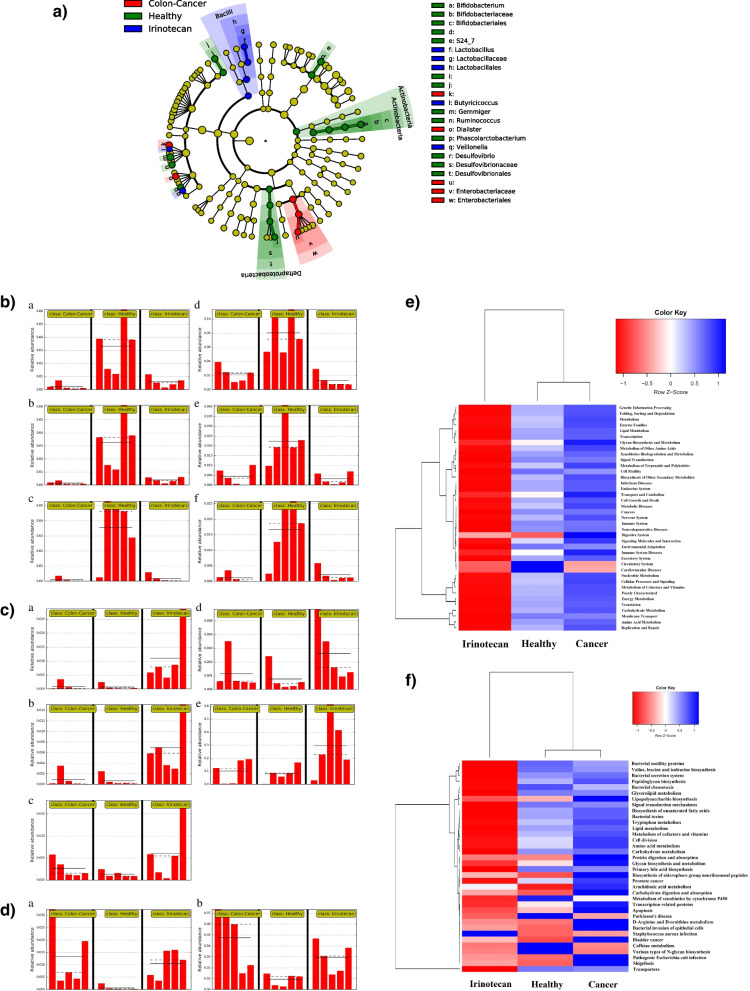
**a**) Linear discriminant analysis (LDA) effect size (LEfSe) of gut microbiota presented in the cladogram; the taxonomic levels are represented by rings. The healthy, colon-cancer, and irinotecan groups are colored in green, red, and blue, respectively; **b**) histogram of relative abundance of significantly noticed gut microbiota in the healthy group—(A, B, C, D, E, and F indicate *Actinobacteria, Bifidobacterium, Bacteroidales S24-7*, *Gemmiger*, *Phascolarctobacterium, Desulfovibronales* respectively); **c**) histogram of relative abundance of marked gut microbiota in Irinotecan group compared to healthy and colon cancer groups—(A, B, C, D, and E indicate *Lactobacillus, Veillonella, Clostridium, Butryicicoccus,* and *Prevotella,* respectively)*;* **d**) histogram of relative abundance of gut microbiota in the colon-cancer group compared to healthy and Irinotecan groups;(A and B indicate *Dialister* and *Enterobacteriaceae*, respectively); **e**) predicted function of the gut microbiota in the Irinotecan, Healthy, and colon-cancer groups according to KEGG pathway hierarchy level 2 [38]. The vertical columns represent groups, and the horizontal rows depict metabolic pathways. The color coding is based on row z-scores; **f**) predicted function of the gut microbiota in the Irinotecan, Healthy, and colon-cancer groups according to KEGG pathway hierarchy level 3 [38]. The vertical columns represent groups, and the horizontal rows depict metabolic pathways. The color coding is based on row z-scores

Linear discriminant analysis (LDA) effect size (LEfSe) analysis uses the Kruskal–Wallis test, Wilcoxon-Rank Sum test, and Linear Discriminant Analysis to find biomarkers of groups. This analysis was able to pick some of the minor taxa distinguishing groups from each other (Fig. 4a). Indeed, there was a decrease in abundance of the phylum *Actinobacteria*, *Verrucomicrobia;* the genus of *Bifidobacterium*, *Gimmiger,* and *Phascolarctobacterium*; and the family of *Bacteroidales S24-7* and *Desulfovibronales* in colon cancer and Irinotecan groups compared to healthy group (Fig. 4b). Especially *Verrucomicrobia* was not present in both colon-cancer and Irinotecan groups compared to the healthy group. By contrast, the genus of *Lactobacillus*, *Veillonella*, *Clostridium*, *Butryicicoccus,* and *Prevotella* abundance was increased in Irinotecan treated groups compared to healthy and colon-cancer groups (Fig. 4c). In addition, the family *Enterobacteriaceae and* genus *Dialister* were more abundant in the colon-cancer group compared to the healthy and Irinotecan treated groups (Fig. 4d).

Furthermore, to consider the microbiome structure and the microbiome function, we used PICRUSt to predict the metagenome profiles based on *16S rRNA* gene sequence data. The results indicated that all pathways were down-repr**e**sented in Irinotecan-treated patients according to KEGG pathway hierarchy level 2 (Fig. 4e) [38]. When deeply investigating selected microbiome functions according to KEGG pathway hierarchy level 3 (Fig. 4f) [38], it was found in the colon-cancer group that certain pathways were overrepresented related to shigellosis, pathogenic *E. coli* infections, bladder cancer, prostate cancer, bacterial invasion to epithelial cells, bacterial toxins and apoptosis. Also, pathways associated with *Staphylococcus aureus* infection were overrepresented in Irinotecan treated group.

### Expression of β-glucuronidase from *E. coli*, a member of the family *Enterobacteriaceae* that predominate in colon-cancer patients, and its expression in response to probiotic treatments

**Fig. 5:**
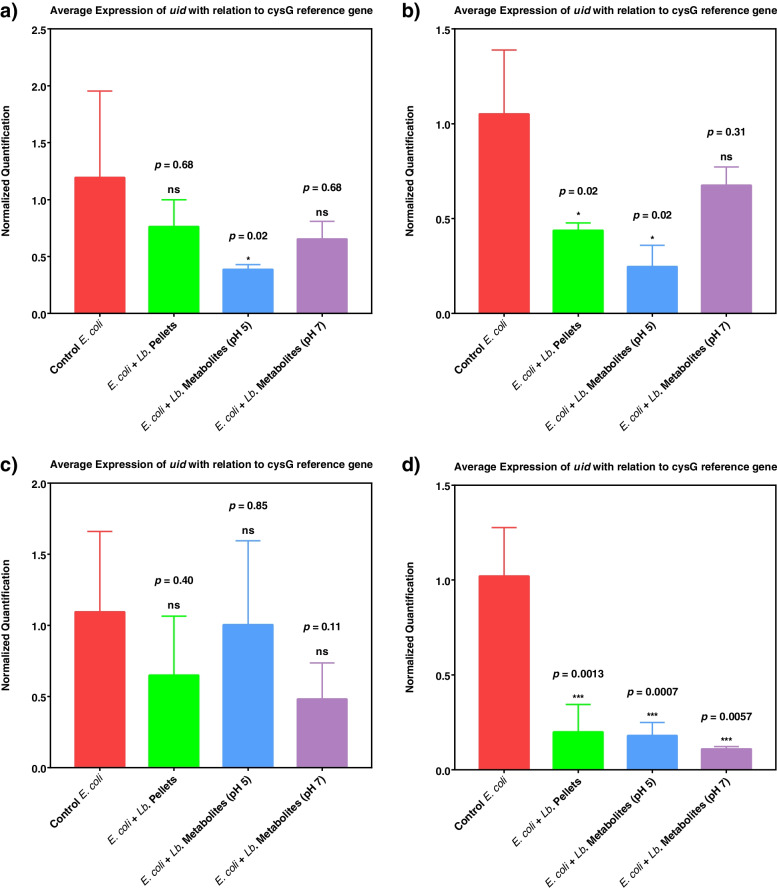
Expression of *uidA* gene (coding for β-glucuronidase) normalized by the expression of *cysG* reference gene when *E. coli* was spiked with; **a**) *L. acidophilus*, **b**) *L. plantarum*, **c**) *L. rhamnosus*, **d**) mixture of three *Lactobucillus* sp. Each experiment was done either using probiotics cells pellets or probiotics raw metabolites in cell-free supernatant adjusted at pH 5 or pH 7. Ns (non-significant) at *p* > 0.05, * *p* < 0.05, ** *p* < 0.01, *** *p* < 0.001

Β-glucuronidase, especially from *E. coli*, participates critically in inducing the diarrheal toxicity of Irinotecan. Here, we investigated the effect of different probiotics or their combination on lowering β-glucuronidase expression (*uidP* gene normalized to *cysG* reference gene). The most significant reduction in *uidP* gene expression was obtained after spiking *E. coli* culture with a mixture of *L. plantarum*, *L. acidophilus,* and *L. rhamnosus* more than using any of them alone (Fig. 5).

### Probiotics in vivo limited Irinotecan-induced oxidative stress

**Fig. 6:**
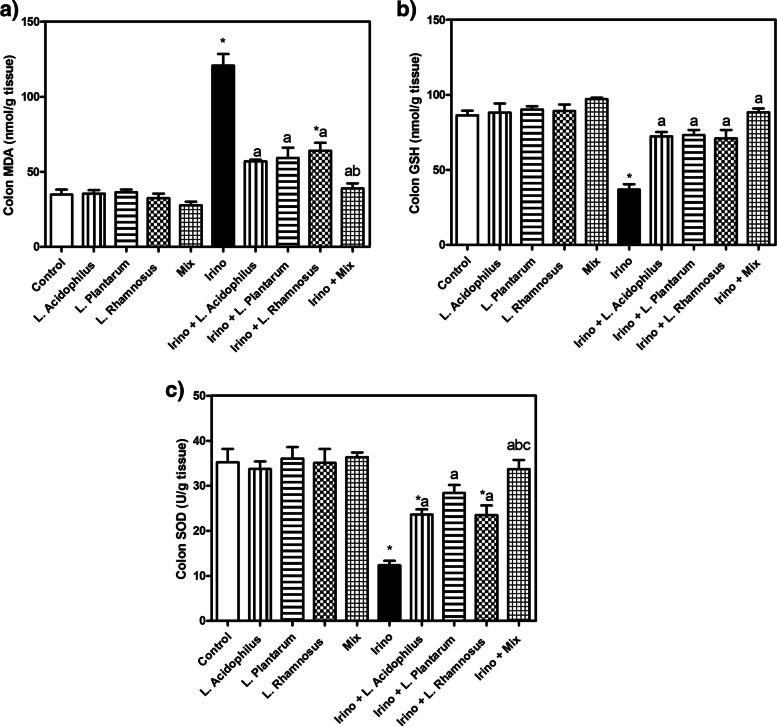
Probiotics limited irinotecan-induced oxidative stress. Colon MDA (A), reduced GSH (B), and SOD (C). Each bar represents the mean ± SEM of 6 mice. Statistics were carried out by one-way analysis of variance (ANOVA) followed by Tukey's multiple comparisons test. *Significant difference from the control group at *p* < 0.05. a Significant difference from the Irinotecan group at *p* < 0.05. b Significant difference from Irinotecan + *L. acidophilus* group at *p* < 0.05. c Significant difference from Irinotecan + *L. rhamnosus* group at *p* < 0.05

An in vivo mice model was used to investigate the ability of probiotics to counteract Irinotecan-induced toxicity. Basically, administration of Irinotecan significantly increased colon MDA content while decreased GSH content and SOD activity compared to the control group. Application of *L. acidophilus*, *L. plantarum*, *L. rhamnosus* and their mixture with Irinotecan evoked a significant decline in colon MDA content with a significant rise in GSH content and SOD activity (Fig. 6A, B and C). The probiotic mixture significantly reduced MDA as compared to *L. rhamnosus* and showed a significant effect on SOD compared to *L. rhamnosus* and *L. acidophilus*. These results together suggest the marked effect of the probiotic mixture that produces better antioxidant effects than using any of them alone.

### Probiotics counteracted Irinotecan-induced inflammatory events

**Fig. 7:**
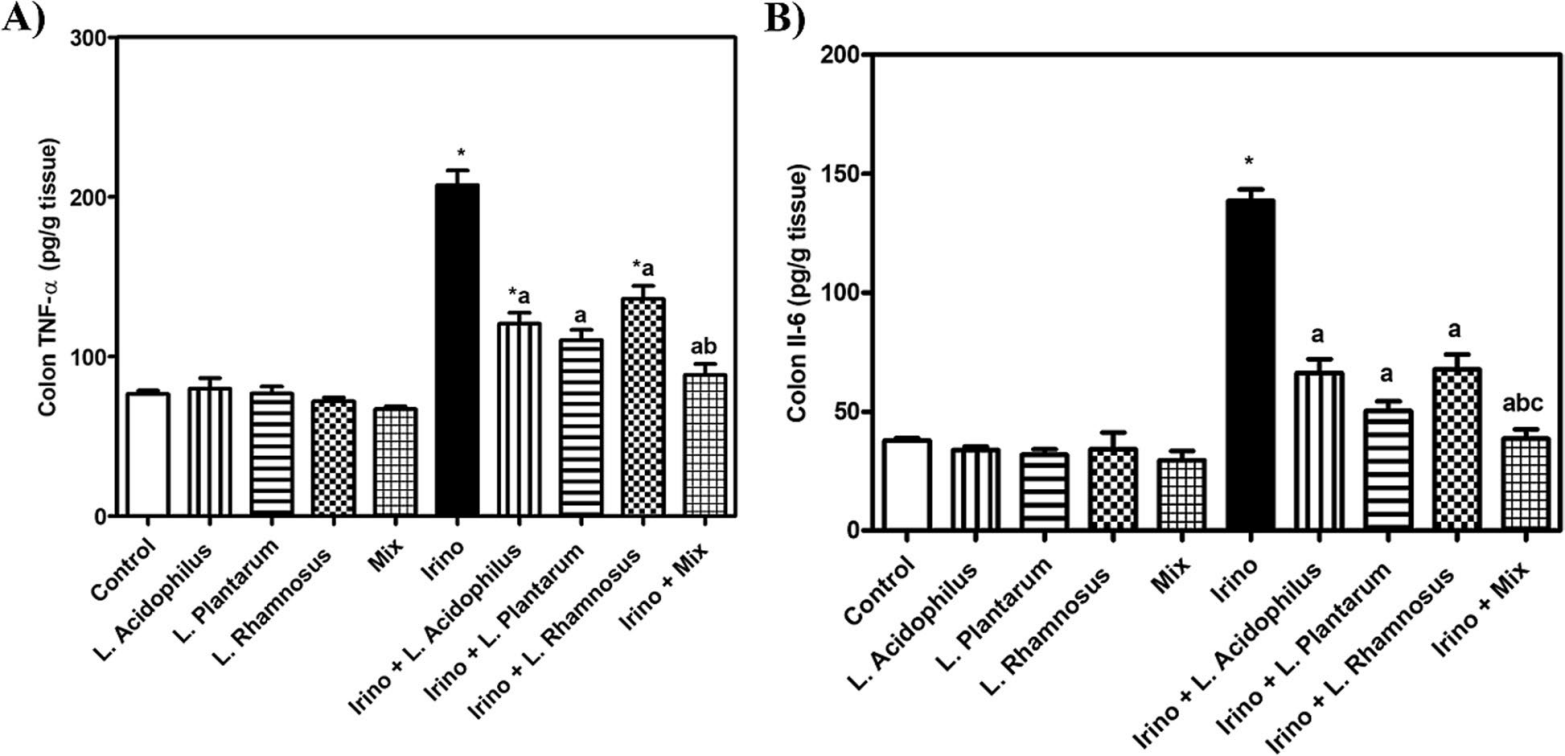
Probiotics counteracted irinotecan-induced inflammatory events. TNF-α (**A**) and IL-6 (**B**). Each bar represents the mean ± SEM of 6 mice. Statistics were carried out by one-way analysis of variance (ANOVA) followed by Tukey's multiple comparisons test. *Significant difference from the control group at *p* < 0.05. a Significant difference from the Irinotecan group at *p* < 0.05. b Significant difference from Irinotecan + *L. acidophilus* group at *p* < 0.05. c Significant difference from Irinotecan + *L. rhamnosus* group at *p* < 0.05. **Figure 8:** Representative photomicrographs exhibited H & E stained colon sections (scale bar, 50 μm); **(A)** Control, showing normal histological architecture. **(B, C, D **and** E)** *L. acidophilus*, *L. plantarum*, *L. rhamnosus* and probiotic mixture treated respectively, showing no histopathological alterations. **(F, G & H)** Irinotecan treated, showing massive inflammatory cells infiltration (if) in the mucosa and submucosa, edema in the submucosa (ed) and focal mucosal necrosis (nc). **(I)** Irinotecan + *L. acidophilus* treated showing apoptosis in the mucosal and glandular epithelium (ap) and submucosal edema (ed). **(J)** Irinotecan + *L. plantarum* showing slight submucosal edema (ed) and few inflammatory cells infiltration (if). **(K)** Irinotecan + *L. rhamnosus*, showing inflammatory cells infiltration in the mucosa (if) and edema in the submucosa (ed). **(L)** Irinotecan + probiotic mixture, showing no histopathological alterations

Administration of Irinotecan significantly increased colon TNF-α and IL-6 protein expression levels compared to the control group. Administration of *L. acidophilus*, *L. plantarum*, *L. rhamnosus,* and their mixture with Irinotecan evoked a significant decline in colon TNF-α and IL-6 protein expression levels compared to the Irinotecan group (Fig. 7A and B). The probiotic mixture significantly reduced colon TNF-α and IL-6 protein expression levels compared to *L. rhamnosus* and *L. acidophilus*. These results propose the marked anti-inflammatory effects of the probiotic mixture rather than using them alone.

### Histopathology

**Fig. 8:**
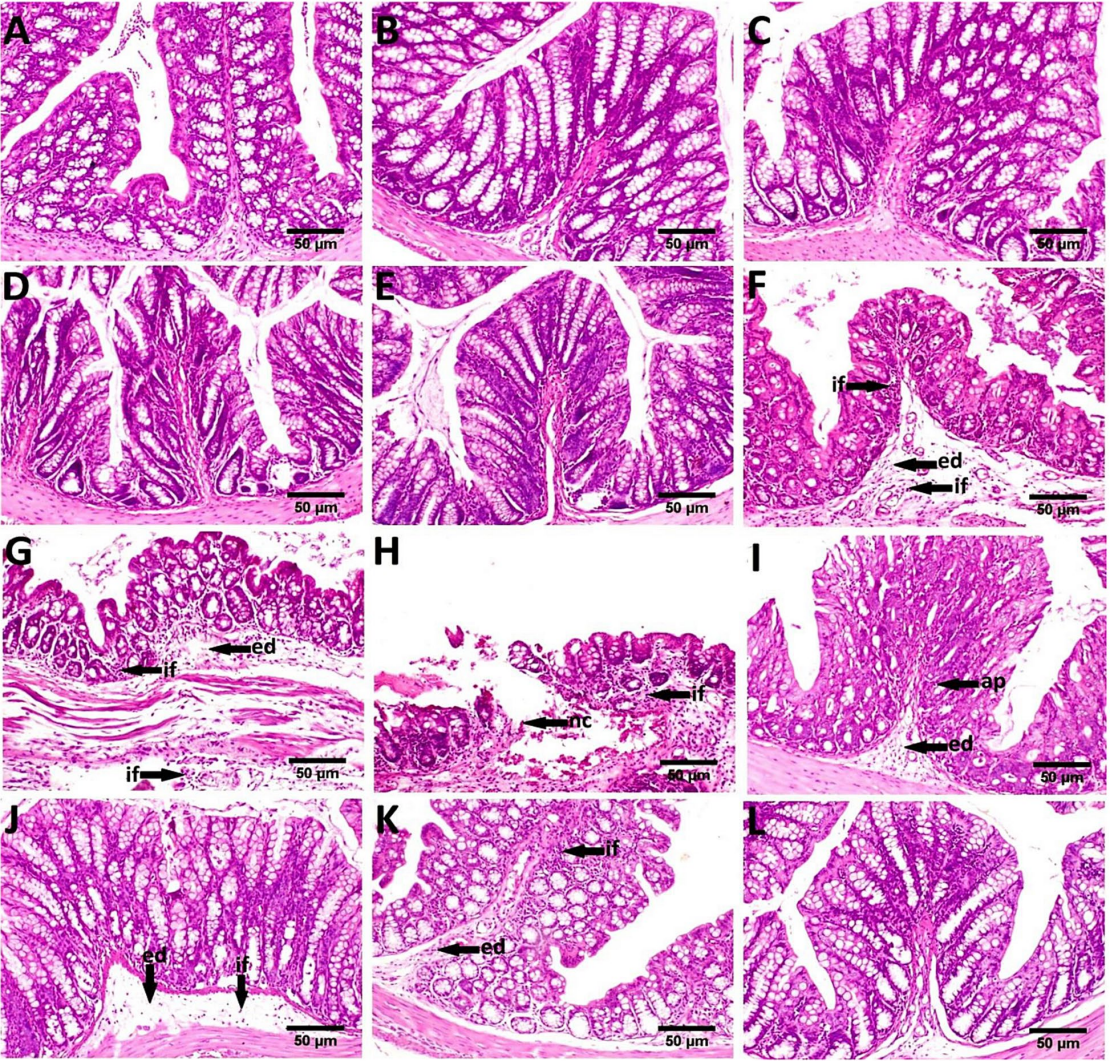
Representative photomicrographs exhibited H & E stained colon sections (scale bar, 50 μm); **(A)** Control, showing normal histological architecture. **(B, C, D **and** E)** *L. acidophilus*, *L. plantarum*, *L. rhamnosus* and probiotic mixture treated respectively, showing no histopathological alterations. **(F, G **and** H)** Irinotecan treated, showing massive inflammatory cells infiltration (if) in the mucosa and submucosa, edema in the submucosa (ed) and focal mucosal necrosis (nc). **(I)** Irinotecan + *L. acidophilus* treated showing apoptosis in the mucosal and glandular epithelium (ap) and submucosal edema (ed). **(J)** Irinotecan + *L. plantarum* showing slight submucosal edema (ed) and few inflammatory cells infiltration (if). **(K)** Irinotecan + *L. rhamnosus*, showing inflammatory cells infiltration in the mucosa (if) and edema in the submucosa (ed). **(L)** Irinotecan + probiotic mixture, showing no histopathological alterations

Microscopically, the colon of control mice showed the normal histological architecture (mucosa, crypts of Lieberkühn, submucosa, and muscularis layers) (Fig. 8A). Furthermore, the colon of mice treated with *L. acidophilus*, *L. plantarum*, *L. rhamnosus,* and probiotic mixtures showed no histopathological alteration (Fig. 8B, C, D and E). In contrast, the colon of mice treated with Irinotecan exhibited severe histopathological lesions described as massive inflammatory cells infiltration in the mucosa and submucosa as well as edema in the submucosa (Fig. 8F and G) and focal mucosal necrosis followed by inflammatory cells infiltration (Fig. 8H). Meanwhile, colon sections of mice treated with Irinotecan + *L. acidophilus* revealed hyperplasia of mucous secreting cells, apoptosis in the mucosal and glandular epithelium as well as submucosal edema (Fig. 8I). On the other hand, improved picture was observed in colon of mice treated with Irinotecan + *L. plantarum*, examined sections showed only slight submucosal edema and few inflammatory cells infiltration (Fig. 8J). Otherwise, moderated improvement was recorded in colon tissue of mice treated with Irinotecan + *L. rhamnosus*, the lesions included, hyperplasia of mucous secreting glands, inflammatory cells infiltration in the mucosa and edema in the submucosa (Fig. 8K). Furthermore, marked restoration of the histological structure of the colon tissue was seen in sections from mice treated with, as examined colon exhibited no histopathological alterations (Fig. 8L). The histopathological lesion scores in the colitis model significantly increased compared to the control and treatment groups. Moreover, the most remarkable improvement was recorded in Irinotecan + probiotic mixtures treated group, as illustrated in (Table 3).

**Table 3:** Histopathological lesion scores

**Group**	**Mucosal necrosis**	**Mucosal inflammatory cells infiltration**	**Edema in the submucosa**	**Submucosal inflammatory** **cells infiltration**	**Apoptosis**
Normal	0 (0–0)	0 (0–0)	0 (0–0.5)	0 (0–0)	0 (0–0)
*L. acidophilus*	0 (0–0)	0 (0–0.5)	0 (0–0.5)	0 (0–0)	0 (0–0)
*L. plantarum*	0 (0–0)	0 (0–0)	0 (0–0.5)	0 (0–0)	0 (0–0.5)
*L. rhamnosus*	0 (0–0)	0 (0–0)	0 (0–0.5)	0 (0–0)	0 (0–0.5)
Mixtures	0 (0–0)	0 (0–0)	0 (0–0.5)	0 (0–0)	0 (0–0.5)
Irinotecan	2 (2–3)^*^	2 (2–3)^*^	3 (3–3)^*^	3 (2–3)^*^	3 (2–3)^*^
Irino + *L. acidophilus*	0 (0–1)	1 (0.5–1)	1 (1–1.5)	1 (0–1)	1 (1–2)
Irino + *L. plantarum*	0 (0–0)^**a**^	0 (0–1)	1 (0–1)	0 (0–1)	1 (0–1)
Irino + *L. rhamnosus*	1 (0–1)	1 (1–2)	1 (1–1.5)	1 (1–1)	2 (1–2)
Irino + *Lb*. Mixtures	0 (0–0)^**a**^	0 (0–0)^**a**^	0 (0–0.5)^**a**^	0 (0–0)^**a**^	0 (0–0.5)^**a**^

Each value represents the median and interquartile range (p25-p75) (*n* = 5). Statistics were carried out by the Kruskal Wallis test, followed by the Dunn test

^*^Significant difference from the control group at *p* < 0.05

^a^Significant difference from the Irinotecan group at *p* < 0.05

### Immunohistochemistry

#### Cleaved caspase-3 expression

**Fig. 9:**
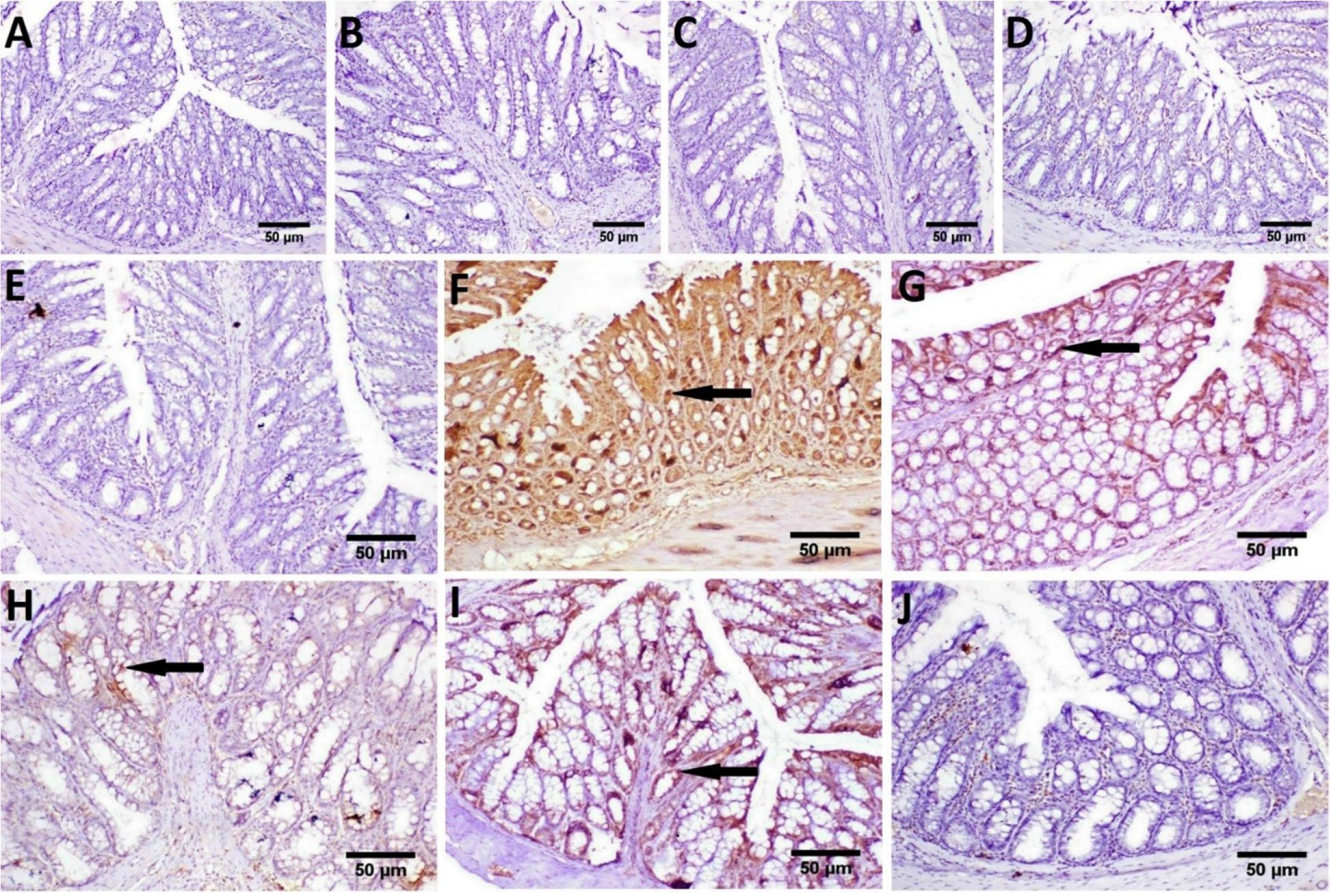
Representative photomicrographs of caspase-3 immune-stained colon sections (scale bar, 50 μm); **A** Control, showing normal no immune expression. **B**, **C**,** D** and** E** *L. acidophilus*, *L. plantarum*, *L. rhamnosus* and probiotic mixture treated respectively, showing no caspase-3 expression. **F** Irinotecan, showing strong positive caspase-3 immune expression (arrow). **G** Irinotecan + *L. acidophilus*, showing a reduced number of brown staining positive cells. **H** Irinotecan + *L. plantarum* showing weak expression of caspase-3 (arrow). **I** Irinotecan + *L. rhamnosus*, showing moderate immune expression (arrow) (**J**) Irinotecan + probiotic mixture, showing no caspase-3 immune expression

**Fig. 10:**
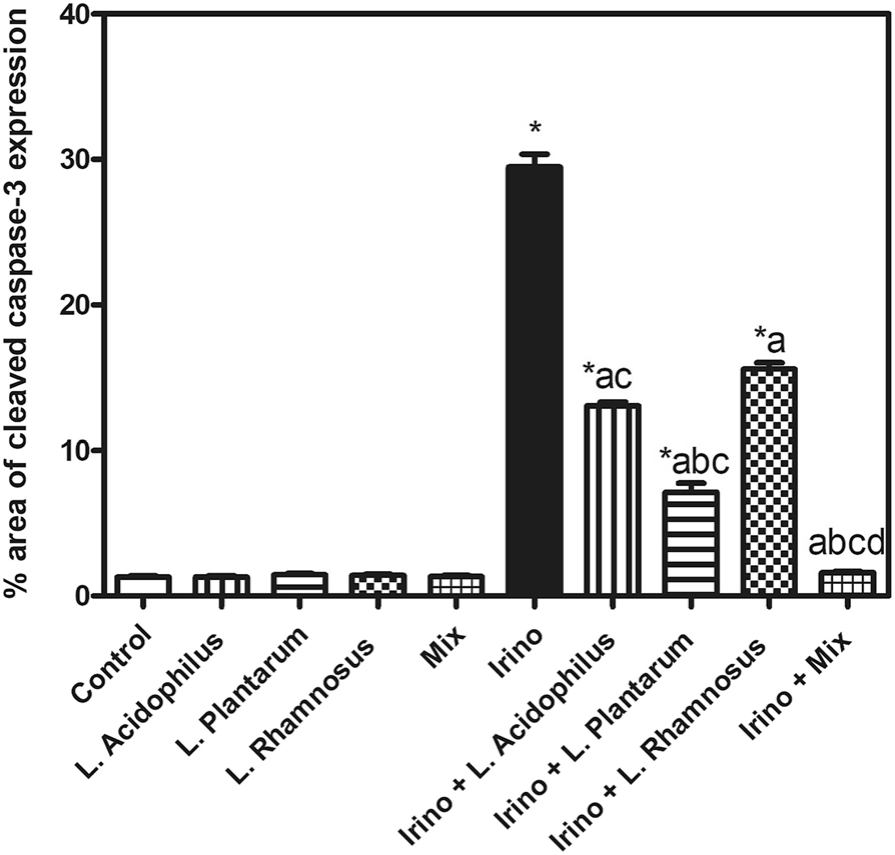
Image analysis results of immunohistochemical investigation of cleaved caspase-3 proteins expression level. Each bar represents the mean ± SEM (*n* = 5). Statistics were carried out by one-way analysis of variance (ANOVA) followed by Tukey's multiple comparisons test. *Significant difference from the control group at *p* < 0.05. a Significant difference from the Irinotecan group at *p* < 0.05. b Significant difference from Irinotecan + *L. acidophilus* group at *p* < 0.05. c Significant difference from Irinotecan + *L. rhamnosus* group at *p* < 0.05. d Significant difference from Irinotecan + *L. plantarum* group at *p* < 0.05

Immunohistochemical staining of caspase-3 revealed no expression in the colon tissue of control normal mice as well as mice treated with *L. acidophilus*, *L. plantarum*, *L. rhamnosus,* and probiotic mixtures (Fig. 9A, B, C, D and E), respectively. On the contrary, a strong positive expression of caspase-3 was recorded in colon sections of Irinotecan-treated mice (Fig. 9F). On the other hand, decreased caspase-3 positive expression was observed in sections from Irinotecan + *L. acidophilus* treated group (Fig. 9G). Meanwhile, weak expression of caspase-3 was exhibited in the colon of mice treated with Irinotecan + *L. plantarum* (Fig. 9H). Otherwise, the colon of mice treated with Irinotecan + *L. rhamnosus* revealed moderate immune expression (Fig. 9I). Conversely, no caspase-3 immune expression was investigated in the colon of mice treated with Irinotecan + probiotic mixtures (Fig. 9J). The image analysis results of immunohistochemical investigation of cleaved caspase-3 proteins expression level are illustrated in (Fig. 10).

## Discussion

Irinotecan (CPT-11) has anticancer activity in various solid tumors. However, severe diarrhea is one of the most common causes of morbidity during Irinotecan-based chemotherapy. Bacterial *β*-glucuronidase is a gut bacterial enzyme that deconjugates SN-38G in the intestinal lumen, releasing the toxic form SN-38, the primary cause of Irinotecan-induced diarrhea. Accordingly, suppressing the bacterial *β*-glucuronidase is considered a prophylactic approach against Irinotecan-induced diarrhea.

In the current study, *L. plantarum*, *L. acidophilus,* and *L. rhamnosus* were investigated in single and mixture forms for their ability to reduce the *β*-glucuronidase expression (*uidP)* from *E. coli*. The most significant reduction in *uidP* was obtained after spiking *E. coli* culture with a mixture of the three *Lactobacillus* spp*.* more than using any of them alone. Although *L. rhamnosus* caused a reduction in *uidP* gene expression, it was not statistically significant. Even though *L. acidophilus* and *L. plantarum* demonstrated statistically significant reductions in gene expression, but the levels were still lower than those induced by the mixture of the three *Lactobacillus* spp. Consequently, the mixture was the best option for reducing β-glucuronidase activity.

Similarly, when *L. plantarum CFR 2194* was co-cultured with *E.coli* in a basic media involving FOS as a carbon source, it inhibited β-glucuronidase to a more significant level, and the production of organic acids in the filtrate, specifically n-butyrate, was indicated to be associated to the detected decline in β-glucuronidase production [39].

Previously, antibiotics including penicillin and streptomycin [40], neomycin [41], and the combination of cholestyramine and levofloxacin [42] were used to lessen the expression of bacterial β-glucuronidase by eliminating microflora. Still, antibiotics exert a harmful effect on other normal beneficial gut microbes. According to the guidelines, delayed diarrhea might be controlled through dietary changes and the use of traditional antidiarrheal drugs based on specific mechanisms, such as somatostatin analog octreotide, loperamide, and deodorized opium tincture. However, these medicines can aggravate pre-existing gastrointestinal problems and induce serious impacts such as irregular heartbeat, respiratory failure, neuropathy, or convulsions [43]. Therefore, it is necessary to find more actual therapies for delayed diarrhea and new emerging methods, including herbal extracts, phytochemicals, and probiotics, to be investigated [43, 44].

To further support the results obtained by the in vitro study, an in vivo study was designed using a mice model to investigate the ability of probiotics to counteract irinotecan-induced diarrhea and decrease its toxicity. The model used in our study was previously reported to mimic mucositis in cancer patients. Irinotecan administration significantly increased colon MDA content while decreasing GSH content and SOD activity compared to the control group. Administration of *L. acidophilus, L. plantarum, L. rhamnosus,* and their mixture with Irinotecan evoked a significant decrease in colon MDA content with a substantial increase in GSH content and SOD activity. The probiotic mixture significantly reduced MDA compared to *L. rhamnosus* and showed a significant effect on SOD compared to *L. rhamnosus and L. acidophilus.* These results together suggest the marked antioxidant effect of the probiotic mixture that produces better effects than the single treatment. Administration of *L. acidophilus****,**** L. plantarum*, *L. rhamnosus* and their mixture did not significantly alter the oxidative stress and antioxidant status of the colon compared to control mice. Malondialdehyde (MDA) is a predictable marker of oxidative stress. Intraperitoneal injection of Irinotecan was reported to increase intestinal MDA content along with a severe reduction in thiol groups, antioxidant protein levels, and activities of antioxidant enzymes, including SOD [45]. In line with the current results, probiotics were found to exert a potent antioxidant effect in diabetic nephropathy via a significant reduction of serum GSH and MDA levels [46].

Similarly, it was reported that prebiotic, probiotic, and synbiotic supplementation significantly increased GSH levels while considerably reducing MDA levels in cardiometabolic and oxidative stress in patients with chronic kidney disease [47]. MDA reduction may be attributed to the improvement of the lipid profile with supplementing probiotics or synbiotics [48]. In addition, probiotics and symbiotics may boost GSH levels by increasing the activity of glutamate-cysteine-ligase (GCL) [49]. Also, *in-vivo* investigations have revealed that different probiotics in the colon environment trigger enhanced SOD production [50].

Histopathological examination of colon sections from Irinotecan-treated mice revealed severe histopathological lesions described as massive inflammatory cells infiltration in the mucosa and submucosa as well as edema in the submucosa and focal mucosal necrosis. These results were confirmed by previously published studies [51–54]. Oral administration of the three *Lactobacillus* spp. revealed potential effect in reducing Irinotecan–induced diarrhea in a schedule-specific manner. Histopathological examination of colon sections of mice treated with Irinotecan + *L. acidophilus* revealed a slightly improved colon picture. On the other hand, there was a marked improvement in examined sections of mice treated with Irinotecan + *L. plantarum.* Also, moderate improvement was recorded in colon tissue of mice treated with Irinotecan + *L. rhamnosus*. Oral administration of *Lactobacillus* spp. mixture showed restoration of the normal histopathological structure of the colon tissue.

Similarly, probiotic preparations such as VSL#3 were previously reported to increase epithelial proliferation and to be included in curing the mucous layer after Irinotecan treatment. Also, VSL#3 reduced intestinal apoptosis following Irinotecan therapy and thus helped to avoid mucosal disintegration and crypt injury. Moreover, VSL#3 prevented the increase in goblet cell counts and mucin secretion after Irinotecan treatment. Such benefits maintain the balance of the water and electrolyte in the gut and prevent diarrhea**.** The protective effects of probiotics are supposed to only be maximal when they are administered in a specific regimen before chemotherapy usage [51].

To further explore the role of inflammation in the toxicity of Irinotecan and the possible anti-inflammatory effect of probiotics as a proposed mechanism for their protective effect, TNF-α, and IL-6 protein expression levels were determined. Irinotecan administration significantly increased colon TNF-α and IL-6 protein expression levels compared to the control group. Administration of *L. acidophilus, L. plantarum, L. rhamnosus,* and their mixture with Irinotecan evoked a significant decreasing in colon TNF-α and IL-6 protein expression levels compared to the Irinotecan group. The probiotic mixture significantly reduced colon TNF-α and IL-6 protein expression levels as compared *L. rhamnosus and L. acidophilus*. These results together propose the marked anti-inflammatory activity of the probiotic mixture than single strains. Oral administration of *L. acidophilus***,** *L. plantarum, L. rhamnosus* and their mixture did not significantly alter the inflammatory status of the colon compared to the control group. To investigate the likely mechanism by which probiotics exert their protective effect, immunohistochemistry labeling of cleaved caspase-3 was performed. Colon sections showed negative expression of cleaved caspase-3 in the colon tissue of control mice as well as mice treated with *L. acidophilus, L. plantarum, L. rhamnosus,* and their mixture.

On the contrary, a strong positive expression of cleaved caspase-3 was recorded in colon sections of Irinotecan-treated mice. Administration of *Lactobacillus* spp. strains either alone or in combination with Irinotecan-treated mice significantly decreased caspase-3 positive expression with better effect shown with the probiotic mixture as proved by nearly normalized caspase-3 expression level. The probiotic treatment prevented apoptosis of epithelial cells in the intestine due to TNF-α, interleukin-1 alpha (IL-1α), and interferon-gamma (IFN-γ) [55]. These pro-inflammatory substances were involved in the prognosis of chemotherapy-induced mucositis [56–58].

In our investigation, we analyzed the action of Irinotecan on the gut microbiota through the in vitro examination of the antibacterial effect of the Irinotecan on the growth kinetics of three *E. coli* isolates and the in vivo effect on the whole gut microbiota. The antibacterial effect of Irinotecan was not investigated in other studies. Irinotecan did not inhibit the growth of *E. coli*; however, it caused a significant delay in *E. coli* growth only at a high concentration of 10 mg/ml. This indicates that gut *E.coli* should still grow and be capable of producing β-glucuronidase, which contributes to Irinotecan toxicity, even in a high concentration of Irinotecan.

We also studied the effect of Irinotecan on gut microbiota via the metagenomic technique by collecting stool samples from healthy people, colon-cancer patients, and Irinotecan-treated patients. The gut microbial community of the three groups was analyzed via a *16S rRNA* sequencing approach. Generally, many factors could affect the gut microbiota, such as the method of delivery, diet, pharmaceuticals, probiotics, prebiotics, fecal transplantation, and demographic factors such as age, sex, and ethnicity [59, 60]. The well-known gut microbiota composition generally relies on four dominant phyla representing more than 90% of the overall microbial communities (*Firmicutes*, *Bacteroidetes*, *Actinobacteria*, and *Proteobacteria*). In addition, it involves other minor phyla such as *Verrucomicrobia* and *Fusobacteria* [61].

In our study, the phyla of *Actinobacteria* and *Verrucomicrobia*, the genus of *Bifidobacterium*, *Gimmiger*, and *Phascolarctobacterium*, and the family of *Bacteroidales* S24-7 and *Desulfovibronales* were highly abundant in the healthy group than in the colon cancer and Irinotecan-treated groups. Reduced abundance of such gut microbiota may be a cause of increased inflammation and a variety of intestinal disorders [62, 63].

It is well known that *Actinobacteria* (for example, *Bifidobacterium*), is one of the predominant phyla of total gut bacteria in healthy people [64]. Moreover, it was reported that the *Verrucomicrobia* phyla, including the genus *Akkermansia,* could increase intestinal levels of endocannabinoids that recover inflammation and gut barrier function [65]. Furthermore, the *Gemmiger* and *Phascolarctobacterium* could synthesize formic, butyric, acetic, propionic, or other short-chain fatty acids (SCFAs) that supply colon cells with about 70 percent of their total energy needs and can be linked to the enhancement of the metabolic state and mood of the host [66].

*Bacteroidales S24-7*, an uncultured family of the order *Bacteroidales*, was shown to be involved in host-microbe interactions that impact gut function and health [67, 68]. *Desulfovibrionales* have a major role in reducing substrates such as taurine into H_2_S, which was reported to be a necessary growth factor for 7α-dehydroxylating beneficial bacteria [69].

In the current study, the colon-cancer group had a greater abundance of the family *Enterobacteriaceae,* and the genus of *Dialister,* but a lower abundance of *Lactobacillus* and *Bifidobacterium* compared to healthy and Irinotecan-treated groups. It is worth mentioning that *Enterobacteriaceae* are frequently regarded as opportunistic pathogens that impair the capability of the gut epithelial layers to do β-oxidation. Accordingly, oxygen is expected to be dispersed in the intestine, promoting colonizing of facultative anaerobic enteric pathogens [70]. Moreover, the initial stages of colorectal carcinoma (CRC) have been characterized by a reduced in microflora such as *Lactobacillus*, *Clostridium,* and *Bifidobacterium*, which are recognized to generate anti-inflammatory SCFAs and were negatively related to the raised markers of injured gut epithelial layers such as diamine oxide (DAO), D-lactate, and LPS. The exclusion of opportunistic infections by commensal bacteria may provide a natural defense against gastrointestinal illnesses, including colon cancer. Probiotic bacteria (*Lactobacillus* spp*.* and *Bifidobacterium* spp*.*) have anti-carcinogenic properties via inactivating microbial enzymes [71].

Along with microbiota dysbiosis, pathogenic bacteria are crucial in some diseases such as CRC. *Prevotella* increased in the colon-cancer group compared to the healthy group in the current study. Higher levels of bacteria belonging to the group *Bacteroides-Prevotella* have been previously noted in stool specimens of patients with CRC compared to healthy controls [72].

Irinotecan treatment apparently caused several microbiota perturbations compared to the healthy and colon-cancer groups, demonstrating increased abundance in the genus of *Lactobacillus*, *Veillonella*, *Clostridium*, *Butryicicoccus,* and even *Prevotella* abundance. Although *Lactobacillus* spp. is predicted to be more abundant in the healthy group, we observed that *Lactobacillus* spp. increased after Irinotecan treatment. This could be attributed to a self-defense mechanism in the host, trying to propagate such beneficial microbes to counteract the diarrheal toxicity of Irinotecan and due to the anti-carcinogenic properties of these bacteria.

The genus *Veillonella* depends on their utilization of pyruvate or lactate, followed by the formation of acetic and propionic acids, H_2_, and Co_2_. Their increase after Irinotecan treatment is because of the increased abundance of lactic acid-producing bacteria such as *Lactobacillus* spp*.* Their rise in numbers immediately accompanies or correlates with the proliferation of lactic acid-producing bacteria [73].

When deeply investigating selected microbiome functions according to KEGG pathway hierarchy level 3, it was found in the colon-cancer group that certain pathways were overrepresented related to shigellosis, pathogenic *E. coli* infections, bladder cancer, prostate cancer, bacterial invasion to epithelial cells, bacterial toxins and apoptosis.

It worth mentioning that toxins and metabolites from some bacteria can influence the DNA stability, cell cycle, cell proliferation, as well as tumor initiation and development [3, 16]. Some bacteria have evolved methods to disrupt DNA in order to eliminate rivals and continue thriving in the microbiome. These bacterial defense factors can unfortunately cause mutations that play a role in carcinogenesis [3].

Several proteobacteria generate cytolethal distending toxin (*CDT*) and colibactin (encoded by the *pks* locus and expressed by *Escherichia coli* [74] and other *Enterobacteriaceae* [75]). *Bacteroides fragilis* toxin (*Bft*) is produced by enterotoxigenic *B. fragilis* [3, 76, 77]. Colibactin has emerged as a molecule of interest in colorectal carcinogenesis, as evidenced by the identification of *pks* + *E. coli* in human colorectal tumors and the potential of colibactin-expressing *E. coli* to exacerbate intestinal tumorigenesis in mice [78, 79]. Mammalian cells are susceptible to double-stranded DNA damage by colibactin and CDT [80]. The host's DNA is damaged indirectly by Bft because it causes a rise in reactive oxygen species (ROS) [81]. High quantities of reactive oxygen species (ROS) can cause DNA damage and mutations by overwhelming the body's natural ability to repair the damage [3].

## Conclusion

Intestinal microbiota was modified by colon-cancer, and by Irinotecan-based chemotherapy. The gut microbiota participates greatly in determining both the efficacy and toxicity of chemotherapies, of which the bacterial ß-glucuronidase enzymes cause the toxicity of Irinotecan. The gut microbiota can now be aimed and modulated to promote efficacy and decrease the toxicity of chemotherapeutics. Probiotics sustain mucosal integrity during chemotherapy usage and can lessen or diminish the toxicity of the Irinotecan chemotherapeutic drug. The use of probiotics to prevent mild inflammation in the colonic mucosa by bacterial transformation would be the most promising future treatment. Oral administration of *Lactobacillus* spp. mixture showed restoration of the normal histopathological structure of the colon tissue. The protective effects of probiotics are only maximum when administered in a specific regimen, namely before and after chemotherapy administration. Probiotics diminish Irinotecan-induced intestinal mucositis through inhibition of inflammation and oxidative harm. This study highlighted how to counteract the Irinotecan toxicity by using probiotics in lowering ß-glucorunidase expression from *E. coli* and hence lowering diarrhea. Finally, this study reported using specified probiotic regimen in lowering mucositis and abnormal histological architecture, lowering oxidative stress of Irinotecan, lowering cellular inflammation of Irinotecan and lowering apoptotic cascade induction of Irinotecan.

The limitation of this study was the reduced number of subjects, the lack of consideration for some variables and the variety of subjects to enroll in the study. Environmental and cultural (and therefore food) differences were not taken into account. Furthermore, the current study does not address the potential role of rare community members, or complex interplay between bacteria, viruses, and fungi.

## Supplementary Information

**Additional file 1: Table S1.** Demographic data of volunteers' groups from which stool samples were collected. **Fig S1.** Abundance profiling of summarized OTUs in healthy, colon-cancer, and Irinotecan groups, as assessed by 16S rRNA metagenomics sequencing using stacked bar plot at different taxonomic levels of classifications; (a) class, (b) order, (c) family, (d) genus, and (e) species levels. **Fig S2.** Core microbiome refers to the set of taxa that are detected in a high fraction of the population in healthy, colon-cancer, and Irinotecan groups at different taxonomic levels of classifications; (a) phylum, (b) class, (c) order, (d) family, (e) genus, and (f) species levels. **Fig S3.** Clustering & SparCC Correlation Network of microbiota in healthy, colon-cancer, and Irinotecan groups. Each node shows (a) one order of bacteria, (b) one family of bacteria, (c) one genus of bacteria, and (d) one species of bacteria. The size of the node corresponds to the log-transformed relative abundance of the microbiota. **Fig. S4.** The Pattern search plot based on SparCC shows top features correlated on (a) phylum level, (b) class level, (c) order level, (d) family level, (e) genus level, and (f) species level. The features are ranked by their correlation, and the blue bars represent negative correlations, while red bars represent positive correlations. The deeper the color (darker blue or red), the stronger the correlation. To the right is a mini heatmap showing whether the abundance of that features is higher (red) or lower (blue) in each group. **Fig. S5.** Phylogeny and abundance based dendrogram of the population in healthy, colon-cancer, and Irinotecan groups at different taxonomic levels of classifications; (a) phylum, (b) class, (c) order, (d) family, and (e) genus levels.

## Data Availability

16S sequence data were submitted to the Sequence Read Archive (SRA) at NCBI and have been assigned accession numbers (SRR19141967 – SRR19141981), (Bioproject: PRJNA836383) and (Biosample: SAMN28159325 – SAMN28159339). The dataset supporting the conclusions of this article is available in the figshare repository https://figshare.com/articles/dataset/Sequences_rar/20056499.
