# Supplementary material for: Irinotecan-gut microbiota interactions and the capability of probiotics to mitigate Irinotecan-associated toxicity
Source: BMC Microbiol. 2023 Mar 2;23:53. doi: 10.1186/s12866-023-02791-3 (PMC9979425; doi:10.1186/s12866-023-02791-3)
Supplement: Supplementary file 1 — Additional file 1: Table S1. Demographic data of volunteers' groups from which stool samples were collected. Fig S1. Abundance profiling of summarized OTUs in healthy, colon-cancer, and Irinotecan groups, as assessed by 16S rRNA metagenomics sequencing using stacked bar plot at different taxonomic levels of classifications; (a) class, (b) order, (c) family, (d) genus, and (e) species levels. Fig S2. Core microbiome refers to the set of taxa that are detected in a high fraction of the population in healthy, colon-cancer, and Irinotecan groups at different taxonomic levels of classifications; (a) phylum, (b) class, (c) order, (d) family, (e) genus, and (f) species levels. Fig S3. Clustering & SparCC Correlation Network of microbiota in healthy, colon-cancer, and Irinotecan groups. Each node shows (a) one order of bacteria, (b) one family of bacteria, (c) one genus of bacteria, and (d) one species of bacteria. The size of the node corresponds to the log-transformed relative abundance of the microbiota. Fig. S4. The Pattern search plot based on SparCC shows top features correlated on (a) phylum level, (b) class level, (c) order level, (d) family level, (e) genus level, and (f) species level. The features are ranked by their correlation, and the blue bars represent negative correlations, while red bars represent positive correlations. The deeper the color (darker blue or red), the stronger the correlation. To the right is a mini heatmap showing whether the abundance of that features is higher (red) or lower (blue) in each group. Fig. S5. Phylogeny and abundance based dendrogram of the population in healthy, colon-cancer, and Irinotecan groups at different taxonomic levels of classifications; (a) phylum, (b) class, (c) order, (d) family, and (e) genus levels. [file 12866_2023_2791_MOESM1_ESM.pdf]

Supplementary file

Irinotecan-gut microbiota interactions and the capability of probiotics to mitigate Irinotecan-associated toxicity

Marwa S. Mahdy[1], Ahmed F. Azmy[1], Tarek Dishisha[1], Wafaa R. Mohamed[2], Kawkab A. Ahmed[3], Ahmed Hassan[4], Sahar El Aidy[5], Ahmed O. El-Gendy[1*]

Table S1: Demographic data of volunteers' groups from which stool samples were collected

	Groups:		
	**Healthy (%)**	**Colon-Cancer (%)**	**Irinotecan (%)**
**Gender:**			
**Male**	80	40	60
**Female**	20	60	40
**Age range:**			
**15-25**	60	0	20
**26-35**	40	40	20
**36-45**	0	60	60
**Occupation:**			
**Working**	80	40	80
**Not working**	20	60	20

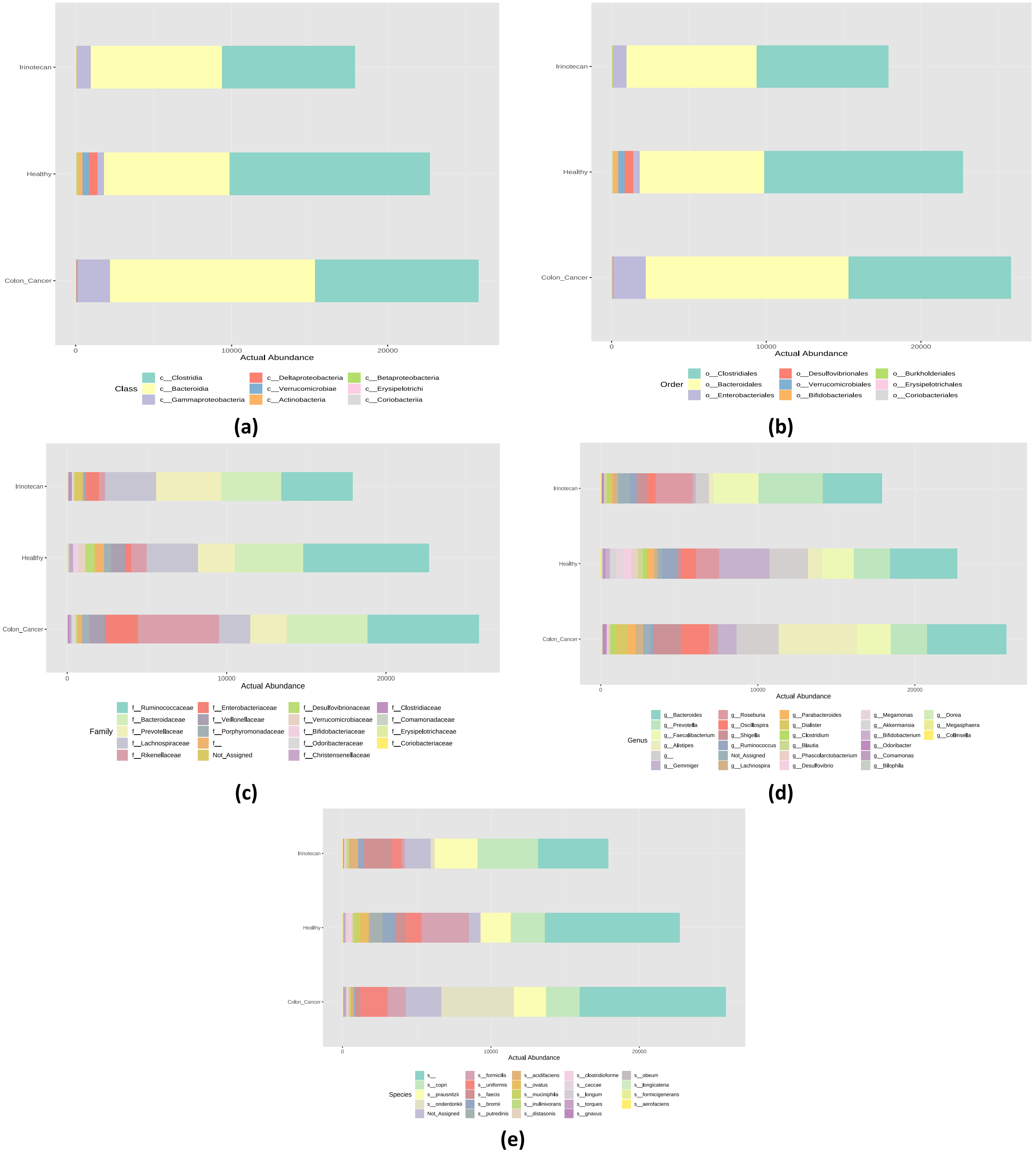

Fig S1: Abundance profiling of summarized OTUs in healthy, colon-cancer, and Irinotecan groups, as assessed by *16S rRNA* metagenomics sequencing using stacked bar plot at different taxonomic levels of classifications; (a) class, (b) order, (c) family, (d) genus, and (e) species levels.

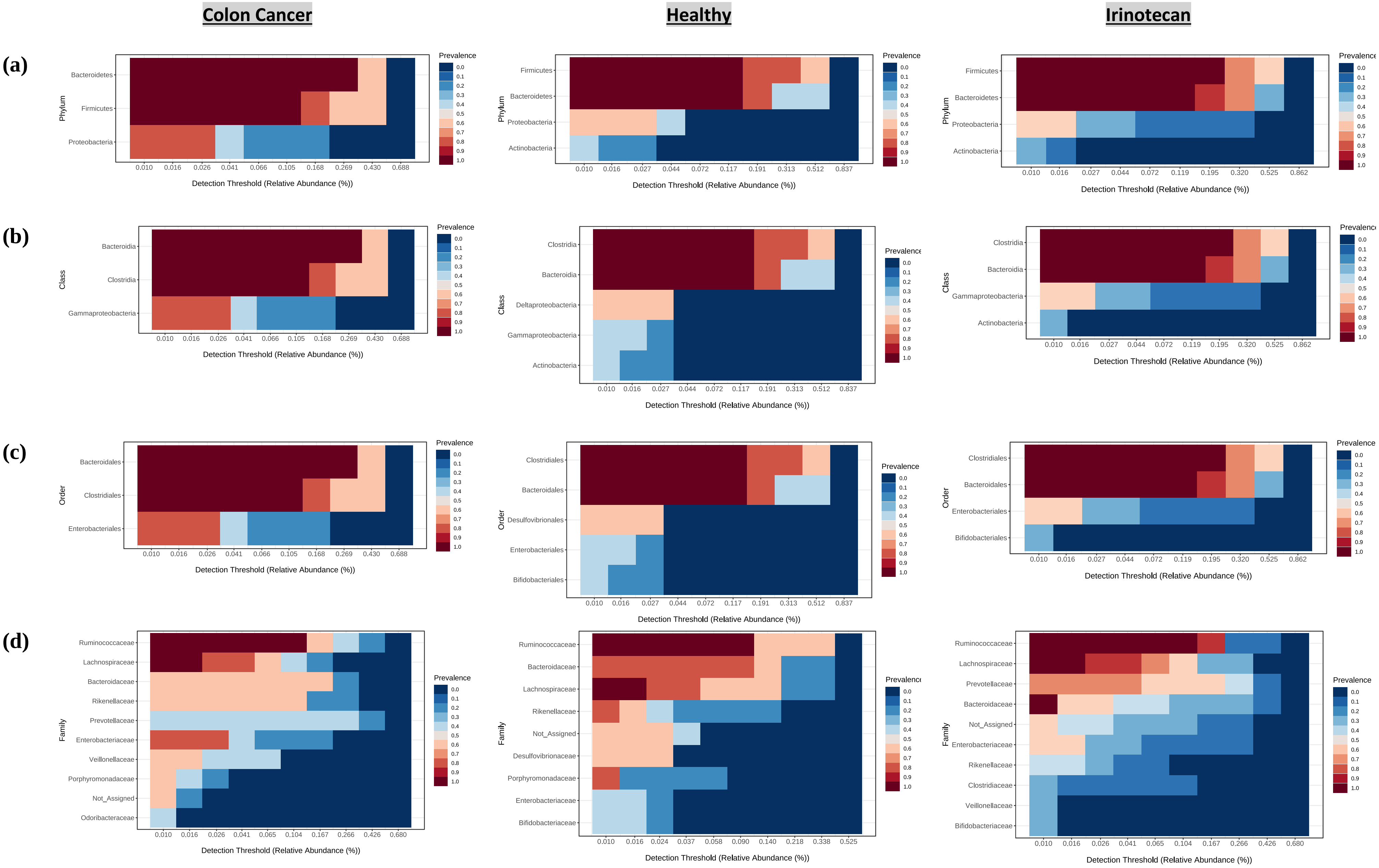

Colon Cancer
Healthy
Irinotecan
(a)
(b)
(c)
(d)
Prevalence
0.0 0.1 0.2 0.3 0.4 0.5 0.6 0.7 0.8 0.9 1.0
Detection Threshold (Relative Abundance (%))
Phylum
Bacteroidetes
Firmicutes
Proteobacteria
Actinobacteria
0.010 0.016 0.026 0.041 0.066 0.105 0.168 0.269 0.430 0.688
0.010 0.016 0.027 0.044 0.072 0.117 0.191 0.313 0.512 0.837
0.010 0.016 0.027 0.044 0.072 0.119 0.195 0.320 0.525 0.862
Class
Bacteroidia
Clostridia
Gammaproteobacteria
Deltaproteobacteria
Actinobacteria
Order
Bacteroidales
Clostridiales
Enterobacteriales
Desulfovibrionales
Bifidobacteriales
Family
Ruminococcaceae
Lachnospiraceae
Bacteroidaceae
Rikenellaceae
Prevotellaceae
Enterobacteriaceae
Veillonellaceae
Porphyromonadaceae
Not_Assigned
Odoribacteraceae
Desulfovibrionaceae
Bifidobacteriaceae
Clostridiaceae
0.010 0.016 0.026 0.041 0.065 0.104 0.167 0.266 0.426 0.680
0.010 0.016 0.024 0.037 0.058 0.090 0.140 0.218 0.338 0.525

(e)

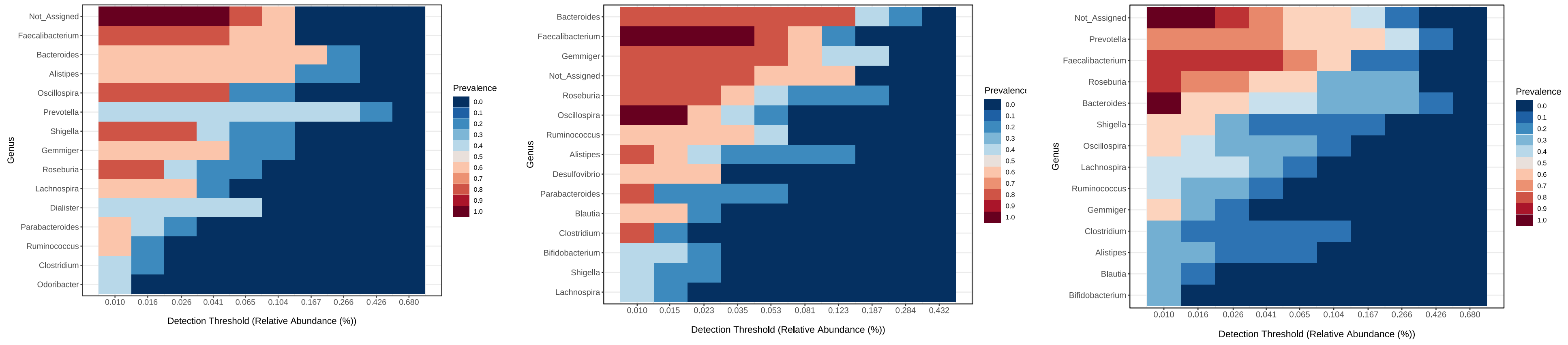

(f)

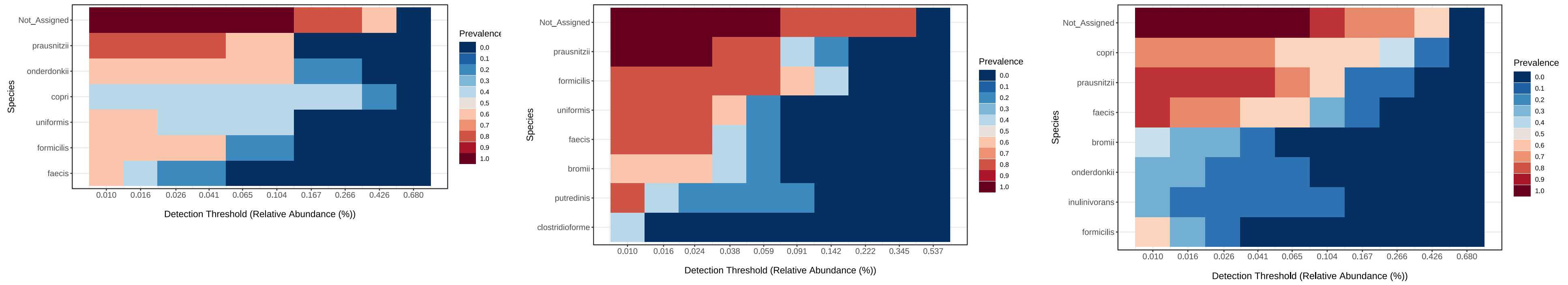

Fig S2: Core microbiome refers to the set of taxa that are detected in a high fraction of the population in healthy, colon-cancer, and Irinotecan groups at different taxonomic levels of classifications; (a) phylum, (b) class, (c) order, (d) family, (e) genus, and (f) species levels.

(a)

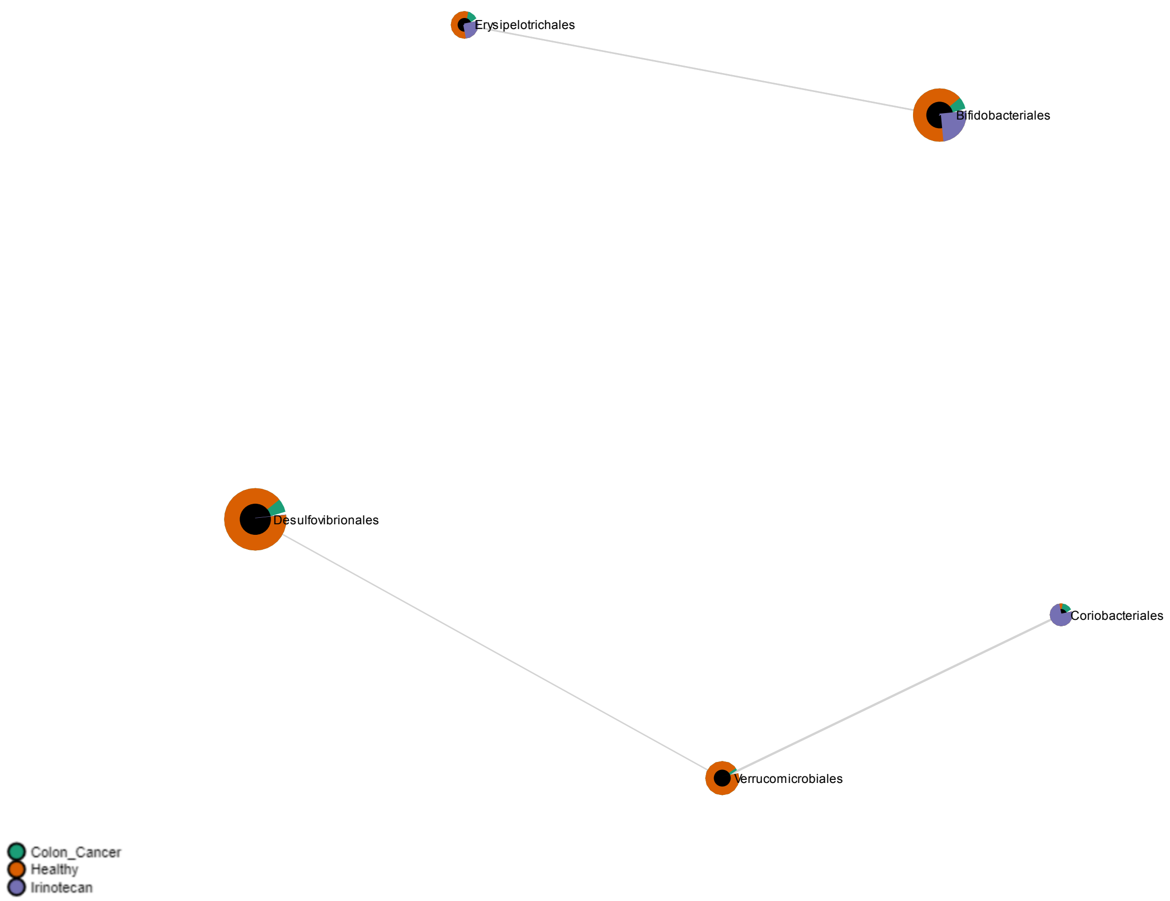

Taxon1	Taxon2	Correlation	*P* Value
Desulfovibrionales	Verrucomicrobiales	0.4352	0.0396
Erysipelotrichales	Bifidobacteriales	0.6952	0.0099
Coriobacteriales	Verrucomicrobiales	1	0.0099

(b)

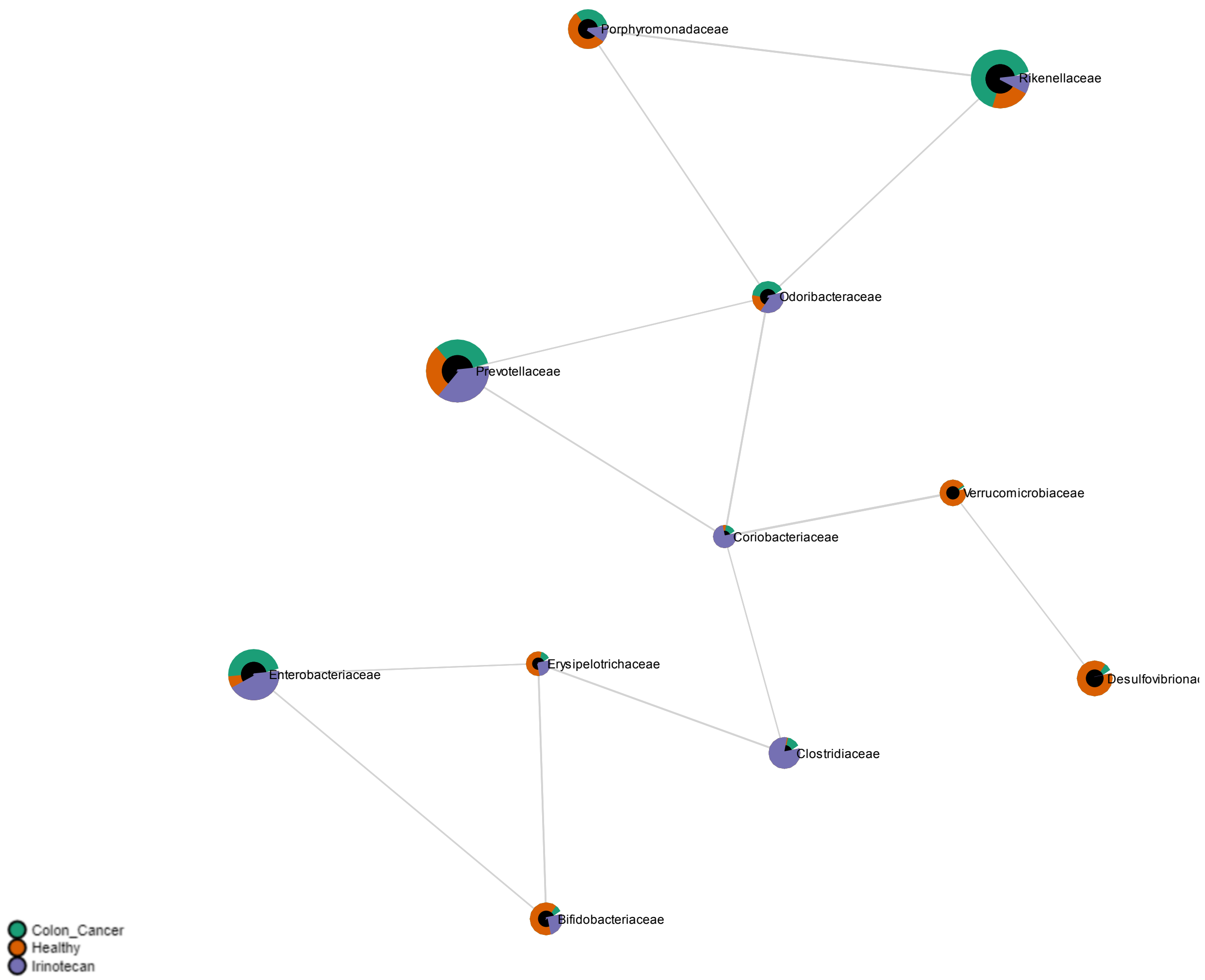

Taxon1	Taxon2	Correlation	*P* Value
Coriobacteriaceae	Verrucomicrobiaceae	-0.7848	0.0198
Odoribacteraceae	Coriobacteriaceae	-0.6757	0.0099
Bifidobacteriaceae	Enterobacteriaceae	-0.5455	0.0297
Erysipelotrichaceae	Enterobacteriaceae	-0.4955	0.0297
Odoribacteraceae	Prevotellaceae	-0.4885	0.0396
Not_Assigned	Enterobacteriaceae	0.4435	0.0396
Not_Assigned	Prevotellaceae	0.4531	0.0495
Coriobacteriaceae	Clostridiaceae	0.4554	0.0396
Desulfovibrionaceae	Verrucomicrobiaceae	0.4676	0.0396
Porphyromonadaceae	Odoribacteraceae	0.5256	0.0297
Odoribacteraceae	Rikenellaceae	0.5335	0.0297
Prevotellaceae	Coriobacteriaceae	0.5915	0.0099
Bifidobacteriaceae	Erysipelotrichaceae	0.6426	0.0099
Erysipelotrichaceae	Clostridiaceae	0.6649	0.0198
Porphyromonadaceae	Rikenellaceae	0.6943	0.0099

(c)

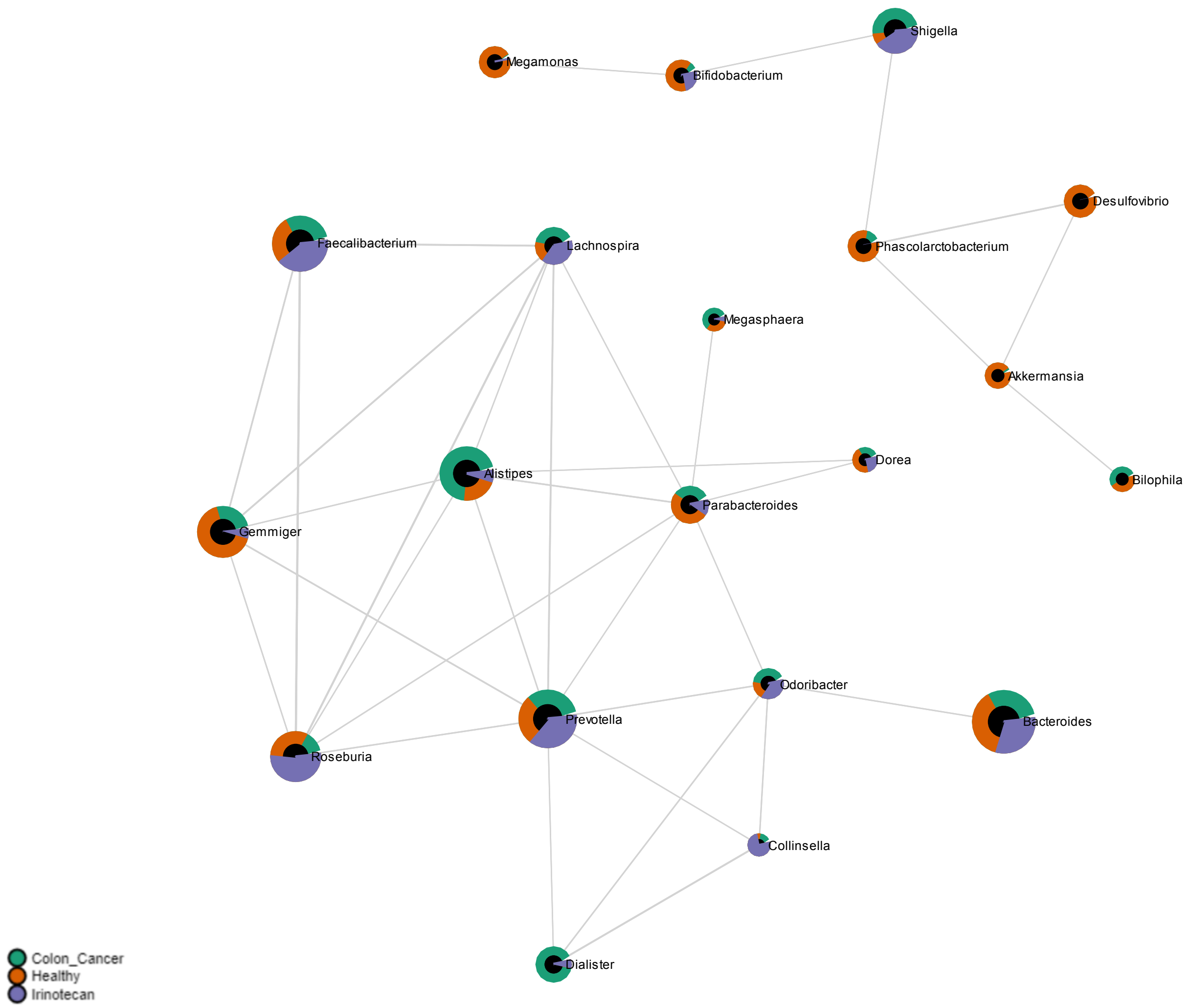

Taxon1	Taxon2	Correlation	*P* Value
Odoribacter	Prevotella	-0.6459	0.0099
Odoribacter	Dialister	-0.6404	0.0099
Collinsella	Odoribacter	-0.622	0.0099
Roseburia	Parabacteroides	-0.6094	0.0297
Alistipes	Prevotella	-0.6079	0.0099
Gemmiger	Alistipes	-0.5894	0.0297
Alistipes	Roseburia	-0.5687	0.0198
Lachnospira	Alistipes	-0.5579	0.0396
Lachnospira	Parabacteroides	-0.5435	0.0297
Shigella	Phascolarctobacterium	-0.495	0.0297
Parabacteroides	Prevotella	-0.4836	0.0396
Shigella	Bifidobacterium	-0.4739	0.0198
Megasphaera	Parabacteroides	0.4264	0.0495
Megamonas	Bifidobacterium	0.4473	0.0198
Akkermansia	Desulfovibrio	0.4503	0.0198
Parabacteroides	Dorea	0.4734	0.0396
Alistipes	Dorea	0.5137	0.0495
Prevotella	Dialister	0.5163	0.0495
Akkermansia	Phascolarctobacterium	0.5258	0.0198
Bilophila	Akkermansia	0.5266	0.0297
Odoribacter	Parabacteroides	0.533	0.0198
Prevotella	Collinsella	0.5649	0.0099
Gemmiger	Roseburia	0.5864	0.0495
Odoribacter	Bacteroides	0.5906	0.0396
Prevotella	Roseburia	0.6343	0.0396
Parabacteroides	Alistipes	0.6988	0.0099
Faecalibacterium	Gemmiger	0.7151	0.0495
Prevotella	Gemmiger	0.7235	0.0099
Desulfovibrio	Phascolarctobacterium	0.7313	0.0099
Prevotella	Lachnospira	0.7694	0.0198
Gemmiger	Lachnospira	0.781	0.0198
Lachnospira	Faecalibacterium	0.7921	0.0396
Collinsella	Dialister	0.8001	0.0099
Roseburia	Lachnospira	0.8538	0.0297
Faecalibacterium	Roseburia	0.9572	0.0396

(d)

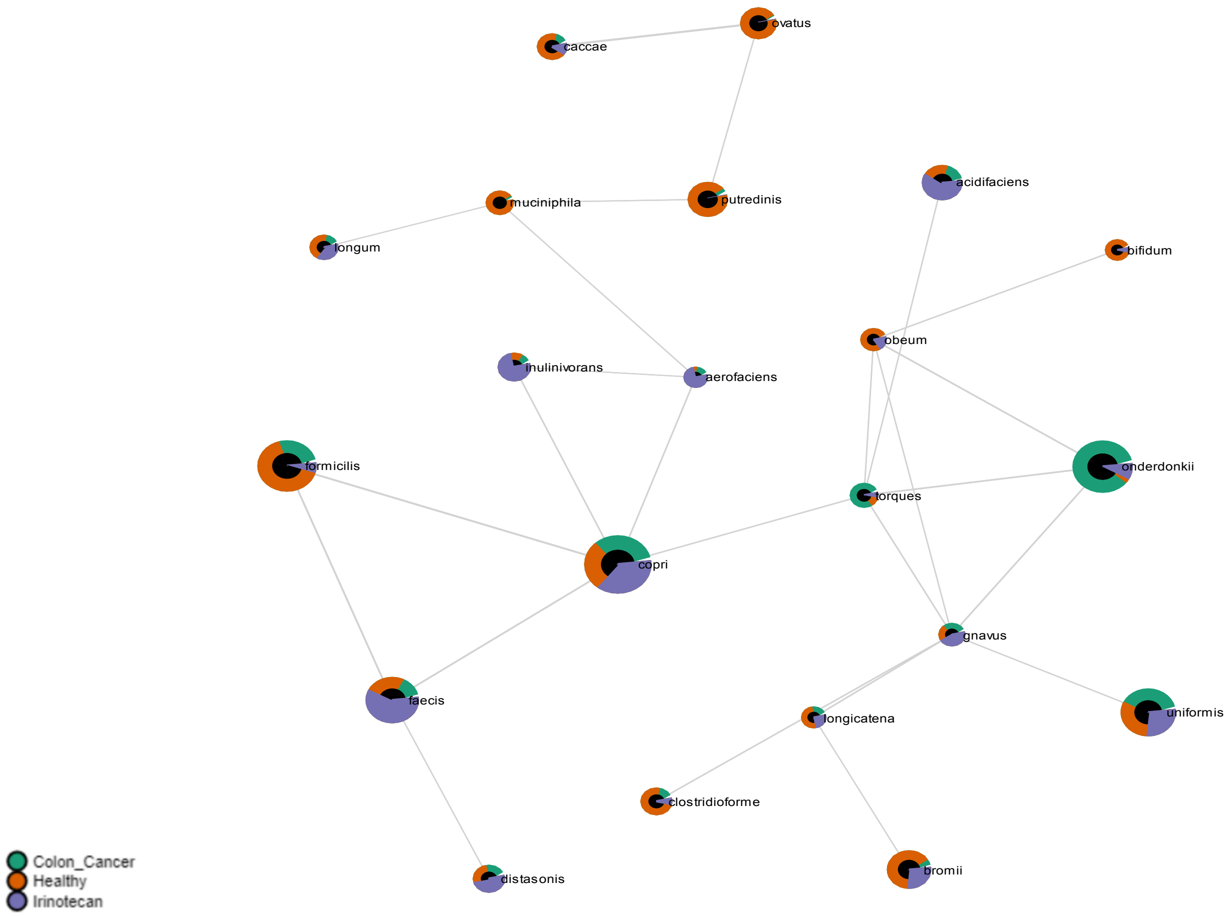

Taxon1	Taxon2	Correlation	P Value
obeum	onderdonkii	-0.6761	0.0099
gnavus	clostridioforme	-0.6039	0.0099
copri	torques	-0.5816	0.0099
muciniphila	aerofaciens	-0.5113	0.0198
obeum	torques	-0.4981	0.0396
obeum	gnavus	-0.4977	0.0297
distasonis	faecis	-0.4926	0.0396
longicatena	bromii	0.3793	0.0495
longum	muciniphila	0.458	0.0495
inulinivorans	aerofaciens	0.477	0.0297
acidifaciens	torques	0.4913	0.0297
putredinis	ovatus	0.4961	0.0297
muciniphila	putredinis	0.5124	0.0396
uniformis	gnavus	0.5472	0.0099
aerofaciens	copri	0.5697	0.0297
torques	gnavus	0.5705	0.0198
bifidum	obeum	0.5719	0.0297
inulinivorans	copri	0.5734	0.0099
gnavus	onderdonkii	0.6375	0.0198
longicatena	gnavus	0.645	0.0099
faecis	formicilis	0.6859	0.0396
torques	onderdonkii	0.6888	0.0099
copri	faecis	0.7126	0.0297
formicilis	copri	0.7933	0.0198
caccae	ovatus	0.8679	0.0099

Fig S3: Clustering & SparCC Correlation Network of microbiota in healthy, colon-cancer, and Irinotecan groups. Each node shows (a) one order of bacteria, (b) one family of bacteria, (c) one genus of bacteria, and (d) one species of bacteria. The size of the node corresponds to the log-transformed relative abundance of the microbiota.

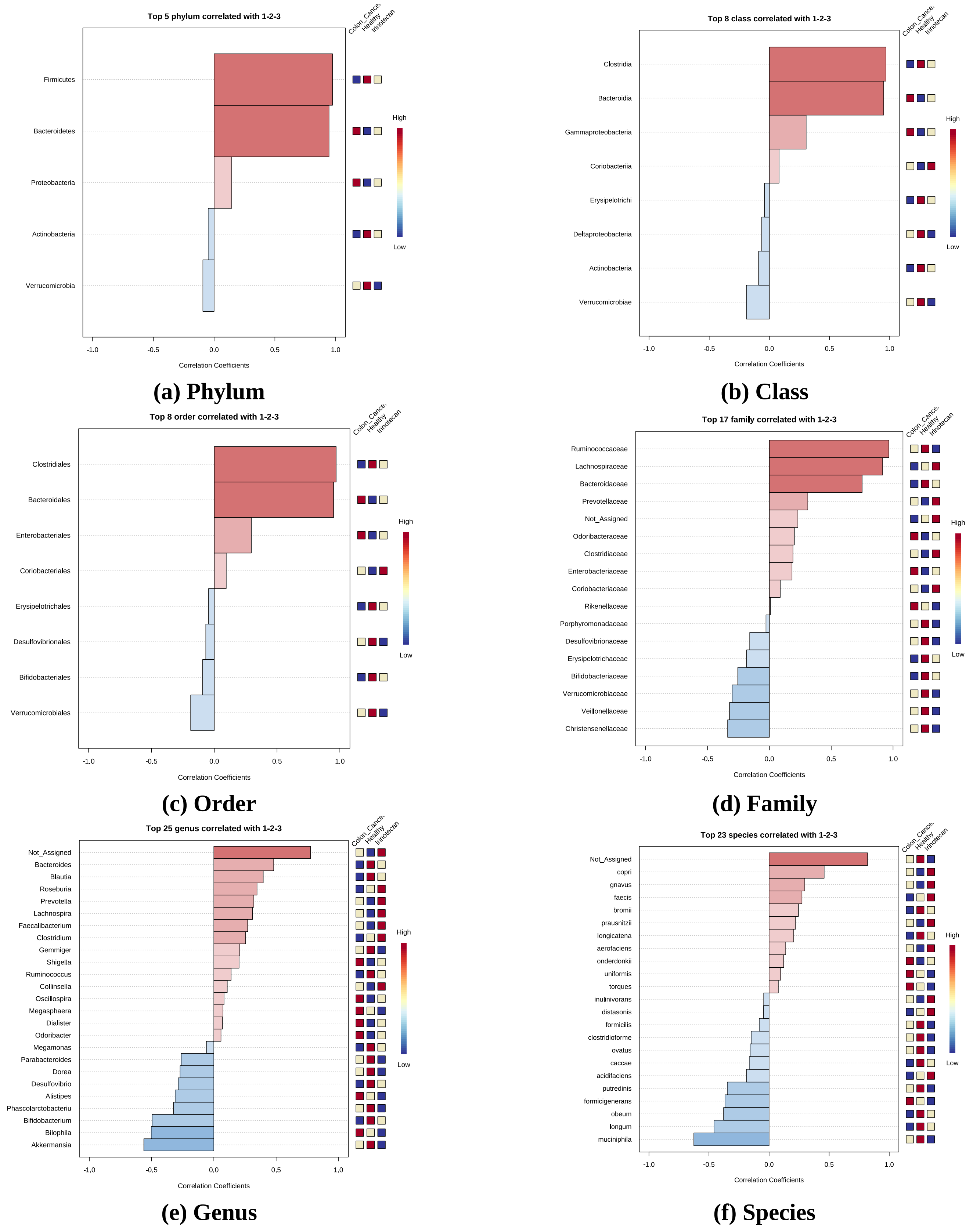

Fig. S4: The Pattern search plot based on SparCC shows top features correlated on (a) phylum level, (b) class level, (c) order level, (d) family level, (e) genus level, and (f) species level. The features are ranked by their correlation, and the blue bars represent negative correlations, while red bars represent positive correlations. The deeper the color (darker blue or red), the stronger the correlation. To the right is a mini heatmap showing whether the abundance of that features is higher (red) or lower (blue) in each group.

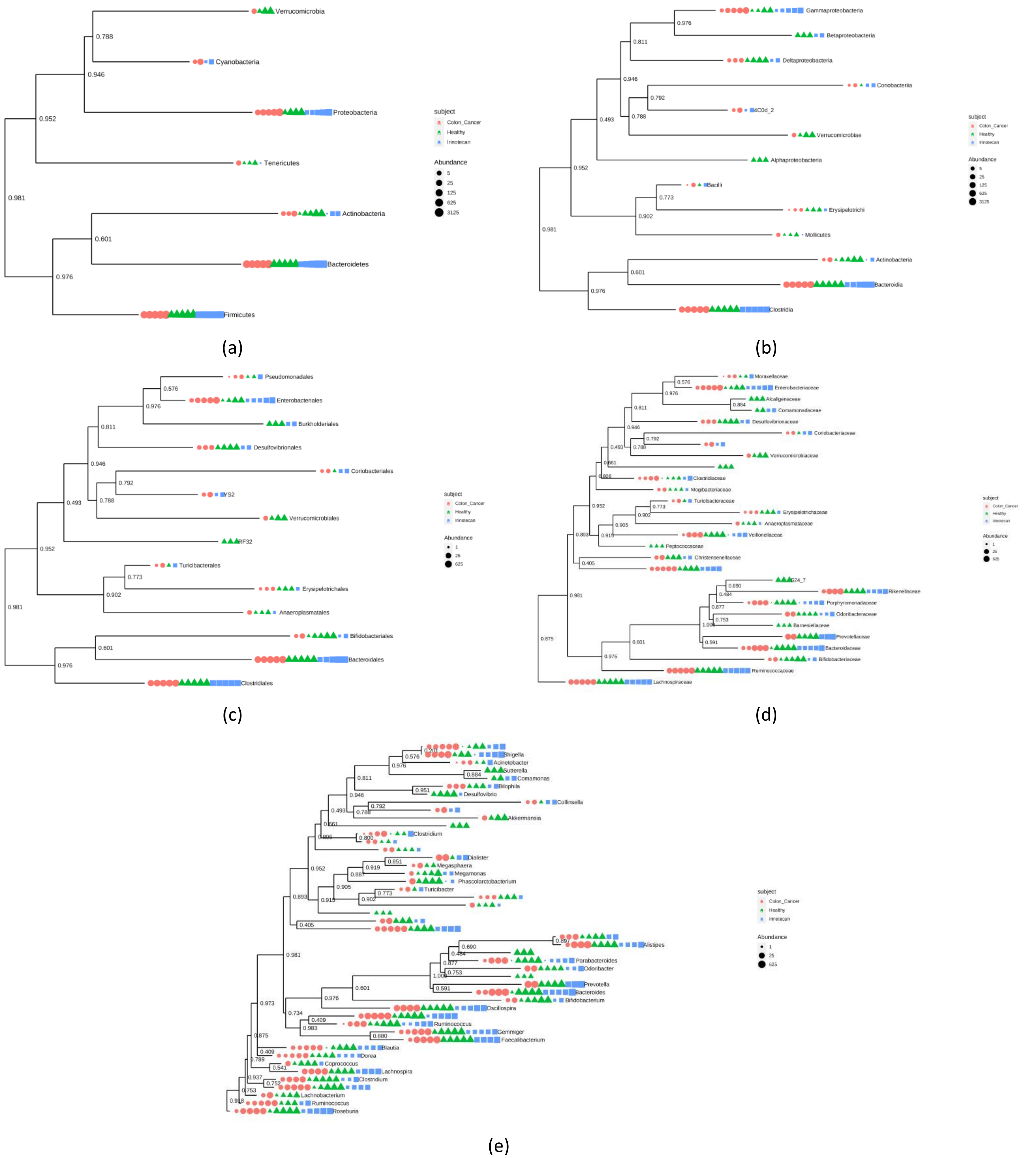

Fig. S5: Phylogeny and abundance based dendrogram of the population in healthy, colon-cancer, and Irinotecan groups at different taxonomic levels of classifications; (a) phylum, (b) class, (c) order, (d) family, and (e) genus levels.
